# Atoll inland and coastal mangrove climate change vulnerability assessment

**DOI:** 10.1007/s11273-022-09878-0

**Published:** 2022-05-11

**Authors:** Nicholas J. Crameri, Joanna C. Ellison

**Affiliations:** School of Geography, Planning and Spatial Sciences, University of Tasmania, Launceston, TAS 7250 Australia

**Keywords:** Marshall Islands, Climate change, Sea level rise, Pacific Islands, Resilience, Wetlands, Inland mangroves

## Abstract

Climate change threatens global mangroves, which are already among the world’s most impacted ecosystems. Vulnerability components of exposure, sensitivity and adaptive capacity were evaluated on mangroves of atoll settings on Jaluit Atoll, in the Marshall Islands, assessing spatial changes of mangrove cover 1945–2018/19, sea-level trends 1968–2019, and reviewing available information. Inland mangrove depressions occur on Jaluit, as well as coastal lagoon margin mangroves, and both were assessed using the same methods. Spatial analysis results showed both inland and coastal mangroves have increased in area. Inland mangroves on eight of Jaluit’s islands mostly expanded after 1976 from 40 to 50 hectares, with progradation and tidal creek infill closing lagoon connections. Shoreline mangroves showed 88–100% of transects prograding 0.1–0.51 m year^−1^ and 0–11.5% of transects eroding 0–0.18 m year^−1^. Assessment of a combination of aerial/satellite images, literature and on-the-ground photos indicated that the mangroves are in healthy condition. Vulnerability assessment results showed both inland and coastal mangroves to have similar strengths and weaknesses in resilience, with intrinsic areas of vulnerability persisting during increased future sea level rise, limited sediment supply and extremely low elevations.

**Supplementary Information:**

The online version contains supplementary material available at 10.1007/s11273-022-09878-0.

## Introduction

Of the impacts of climate change, sea level rise (SLR) is one where devastation of social, economic and environmental systems is considered likely (Dasgupta et al. 2009). This is because of the large amount of infrastructure located near sea level, and so many people having huge economic and nutritional reliance on the ocean (Béné 2006). Recent estimates suggest up to 190 million people live below levels of SLR predicted by 2100, even when applying low emissions projections to models (Kulp and Strauss 2019). Asia, Small Island Developing States (SIDS) and increasingly Africa are especially at risk (Dasgupta et al. 2009; Neumann et al. 2015; Palinkas 2020). In the latest citable Intergovernmental Panel on Climate Change (IPCC) report ‘*Special Report on the Ocean and Cryosphere in a Changing Climate’*, global sea levels are projected to rise by 0.43–0.84 m by 2100 relative to 1986–2005 (Oppenheimer et al. 2019). These estimates are likely to be surpassed with any rapid increase in the melting of polar ice sheets and glaciers (Smith et al. 2020), and in local and regional contexts these projected increases may be even more pronounced (Kopp et al. 2015; Nerem et al. 2018). Such increases in sea level will greatly affect coastal environments, through increased flooding and inundation, erosion and salinity of soils and fresh water supplies (Kirwan and Megonigal 2013; Kopp et al. 2015). Mangroves are a coastal ecosystem particularly vulnerable to SLR (Ellison 2015; Oppenheimer et al. 2019), and are already threatened globally by direct human impacts, despite their many ecosystem services (Duke et al. 2007).

The IPCC adopted a conceptual framework for vulnerability assessments utilising three dimensions: external exposure of systems to change, sensitivity to impacts, and adaptive capacity to accommodate changes (Adger 2006; Ellison 2015; Bueno-Pardo et al. 2021). Analysis of vulnerability indicates how changing conditions affect ecosystems and species (Watson et al. 2017) allowing strategic actions which reduce the risk of threats (Bevacqua et al. 2018). Mangrove vulnerability assessments of components of exposure, sensitivity and adaptive capacity have allowed targeted recommendations in Tanzania, Fiji and Cameroon (Ellison 2015), China, Madagascar, Mozambique, Mexico, Iran and India (Rakotondrazafy et al. 2014; Li et al. 2015; Lee et al. 2018; Charrua et al. 2020; Cinco-Castro and Herrera-Silveira 2020; Majumdar et al. 2021; Mafi-Golami et al. 2021). Spatial analysis using GIS can allow vulnerability assessment, where ground survey is difficult, time consuming or expensive, combined with interviews of environmental experts (Datta 2010; Omo-Irabor et al. 2011).

Low-lying islands, including the atolls of the Pacific, are at great risk to the impacts of climate change, particularly SLR (Thaman 2018). Governments of such nations are aware of threats from storm surges and extreme tides, with these risks factored into national conservation and security planning (Baker et al. 2011; Thaman 2018). Coastal flooding events are estimated to increase in future up to 1000-fold for even the most modest SLR scenarios (Taherkhani et al. 2020). With most atolls only a few meters above sea level at most, complete inundation is a distinct possibility in the near future (Thaman 2018). SLR can cause the intrusion of saline water into critical fresh-water lenses that sustain terrestrial ecosystems and agriculture within atolls (Baker et al. 2011). Many atolls have inland as well as lagoon-fringing mangrove forests, providing similar benefits to other mangroves across the equatorial regions of the world (Woodroffe 1987). Their intrinsic biodiversity values are unique, each Pacific Island group having a differing complement of mangrove-related flora and fauna that decreases in species numbers from west to east across the Pacific along with increasing isolation of habitats (Ellison 2009). On the high island of Viti Levu, Fiji, vulnerability assessment of mangroves to climate change components found higher risk owing to relative sea level rise (RSLR) rates, low sediment supply and accretion rates, and microtidal range (Ellison and Strickland 2015; Ellison 2015). Vulnerability of atoll mangroves to climate change has not been previously considered.

Mangrove sediment supply is important for mangrove resilience to the SLR scenarios that are predicted, and sources differ according to the physiographic settings of mangroves (Di Nitto et al. 2014). For high islands and continental margins, sources include allochthonous fluvial minerogenic sediment, and mangrove systems with such sediment supply have higher net sedimentation rates which makes them less vulnerable to SLR impacts (Lovelock et al. 2015; Ellison 2019). Where minerogenic sediment is low or absent, mangrove sediment accretion derives from biogenic sources such as root mat development (McKee et al. 2011). Atolls, 436 in total globally (Goldberg 2016), have very low elevations and rely solely on sediments derived from coral reefs and associated molluscs, calcareous algae and foraminifera (Ellison et al. 2019). This study investigates the mangroves of Jaluit Atoll located in the Republic of the Marshall Islands (RMI) in Micronesia. Jaluit is one of 27 atolls in the RMI, and mangroves are previously little studied in terms of their spatial extent, characteristics and vulnerability to climate change (Bungitak 2003; Thaman 2018). Jaluit is a Ramsar designated Wetland of International Importance, one of only two existing sites in the RMI (Bungitak 2003). One of the intentions of this study is to assist in the update of the Ramsar Information Sheet for Jaluit Atoll, thus enabling ongoing management of this important wetland.

The research questions of this study are:

1. What is the current vulnerability of Jaluit Atoll mangroves to sea-level rise?
2. How are inland and coastal mangroves of atolls similar or different in their vulnerabilities?

## Methods

### Study site

**Fig. 1:**
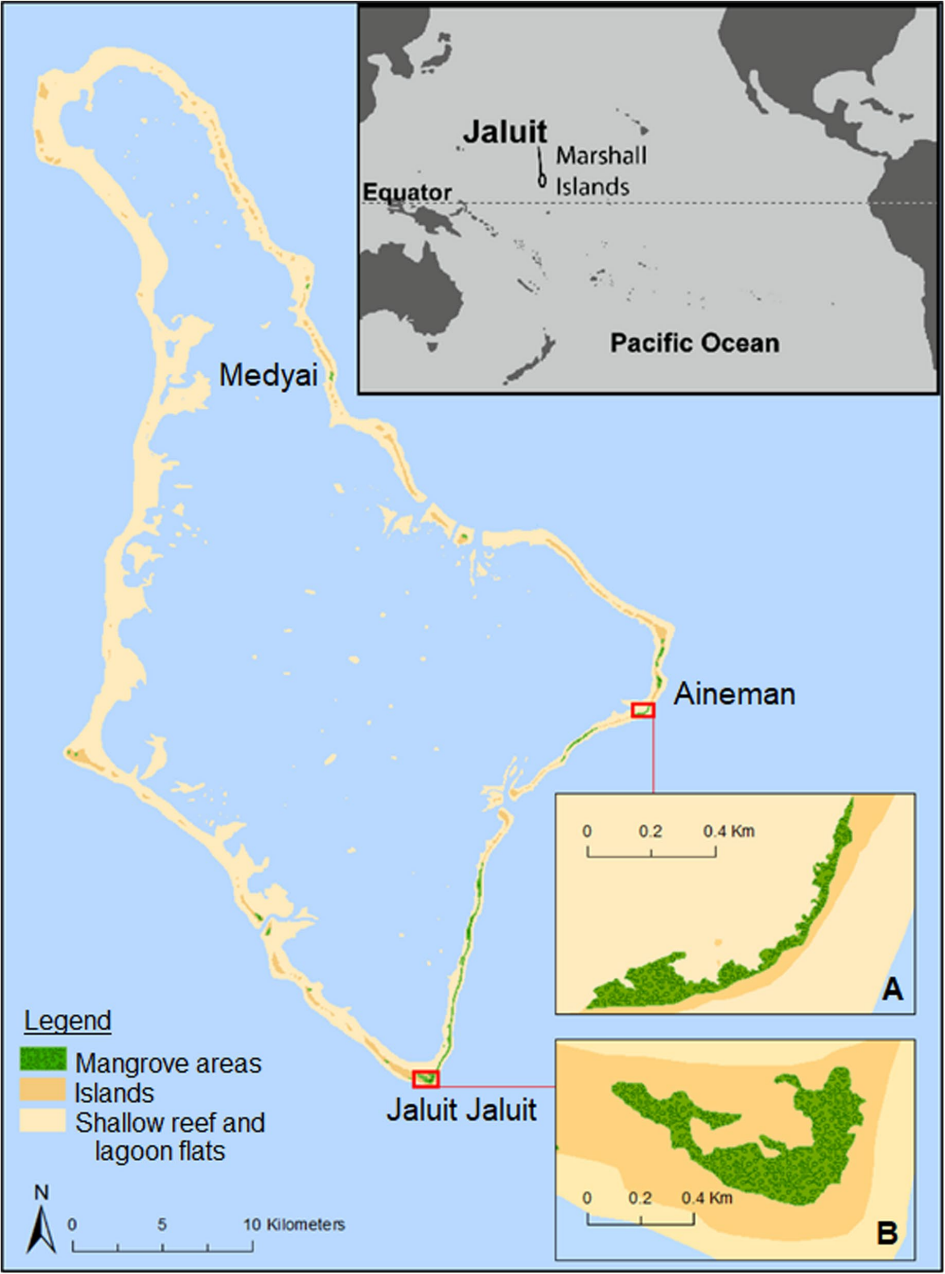
Location of Jaluit Atoll and examples of mangrove areas **A.** Lagoon fringing mangroves on Aineman island and **B.** Inland mangroves on Jaluit Jaluit island

Jaluit Atoll (Fig. 1) (6.0° N; 169.35° E) is part of the Ralik Island Chain and contains 91 islands (Ford and Kench 2016). It is located approximately 200 km south-west of Majuro, the capital of the RMI (Lindsay and Aiello 2003). The atoll is ~ 60 km long and ~ 34 km wide (Ford and Kench 2016). While the total land area is only 11.34 km^2^ (Kench et al. 2015), the area of the entire atoll is 689.74 km^2^ (Ford and Kench 2016; SPREP 2016), the majority being the atoll lagoon. Annual rainfall is ~ 3400 mm (NOAA 2020b), relative humidity mostly 75–85% (SPBCP 1999) and average air temperature is 26–28 °C (Deunert et al. 1999). The total population is 1788 (Economic Policy Planning and Statistics Office and SPC 2011), and major economic activities are commercial fisheries and ecotourism (Lindsay and Aiello 2003).

Jaluit was designated as a Ramsar Wetland of International Importance in 2004 (Bungitak 2003), and subsequently no further update of information on the Ramsar Information Sheet has occurred. It is a low-lying atoll, with six mangrove species identified. Jaluit possesses globally significant ecosystems in its coral reefs, lagoons and mangrove areas, and while most Pacific Islands have limited mangrove species present, Jaluit has a relatively high number of species for an atoll biome, with both inland and shoreline mangroves. In addition, internationally threatened species such as green (*Chelonia mydas*) and hawksbill sea turtles (*Eretmochelys imbricata*), and the giant clam (*Tridacna gigas*) add to its significance (Lindsay and Aiello 2003).

Mangrove species identified on Jaluit are *Bruguiera gymnorhiza*, *Sonneratia alba*, *Rhizophora apiculata*, *Lumnitzera littorea*, *Pemphis acidula*, and *Xylocarpus cf. rumphii*. (Fosberg and Sachet 1962; Deunert et al. 1999; Vander Velde and Vander Velde 2005). Mangrove associates identified are *Asplenium nidus* and *Nephrolepis acutifolia* (Fosberg and Sachet 1962). Of the mangrove species on Jaluit, Fosberg and Sachet (1962) described *Lumnitzera littorea* as occurring in mangrove depressions, *Bruguiera gymnorhiza* as abundant in swamps, along with *Sonneratia alba*. *Pemphis acidula* grows on rocky, saline areas (Fosberg and Sachet 1962), yet *Pemphis* is classified as a true mangrove species by Polidoro et al. (2010). *Xylocarpus* species of *X. rumphii* and *X. granatum* are very similar species, and are common in landward mangrove locations in the region (Duke et al. 2012).

Two types of mangrove areas occur on Jaluit, inland mangrove depressions (Fosberg 1975; Woodroffe 1987), and coastal mangroves on the leeward lagoon shore of the atoll islands. Inland mangrove depressions have hard-bottomed coral limestone basements, and are located inland on atoll islets, which is a common setting for mangroves in the Marshalls. These depressions are fed by seepage of groundwater, somewhat saline (Fosberg 1975). There is little information published on the coastal mangroves of Jaluit, though Fosberg (1975) notes a greater development of mangrove swamps relative to other islands in the Marshalls.

### Vulnerability assessment methods

Coastal vulnerability assessments can allow quantitative analysis of both social and biophysical vulnerability using interdisciplinary methods, to allow prioritization strategies (Bevacqua et al. 2018). The three dimensions of vulnerability (exposure, sensitivity and adaptive capacity) are individually not quantitative, but components of each dimension can be measured using established techniques (Ellison 2015). Exposure components assessed were relative sea level trends, tidal range, geomorphic setting, and climate (rainfall) modelling (Table 1). Sensitivity components assessed were mangrove spatial changes, seaward edge retreat, mangrove condition, recruitment, sedimentation rates, adjacent seagrass and coral reef condition. Adaptive capacity components assessed were available migration areas, mangrove protection status, local management capacity, and stakeholder involvement. SIDS of the Pacific widely closed borders and imposed strict internal measures following the COVID-19 outbreak in early 2020, hence the vulnerability assessment was adapted to focus on remote sensing and review methodologies. Given the paucity of scientific assessment information available for environmental understanding in the Pacific SIDS, a non-conventional approach was used to gather information for this study, including non-academic sources of information. Searches of governmental databases from the Marshall Islands, the United States of America and Australia were conducted for information on climate, sea levels and tide information. Searches were furthermore conducted on the databases of international organizations such as the Ramsar Convention and Secretariat of the Pacific Regional Environment Programme (SPREP) and the University of the South Pacific Library Pacific Collection for specific information on Jaluit Atoll. Also, academic databases including Google Scholar, Web of Science and Scopus were accessed and searches included but were not limited to ‘Marshall Islands mangroves’ OR ‘Jaluit Atoll mangroves’ OR ‘mangroves remote sensing’ OR ‘Marshall Islands environmental protection’.

**Table 1:** Vulnerability assessment components evaluated ( adapted from Ellison 2015)

Dimension of vulnerability	Vulnerability assessment component	Approach
Exposure	Relative sea level trends	Tide gauge analysis (PSMSL 2020)
	Tidal range	Local tidal information
	Sediment supply type	Assessment of geomorphic setting
	Climate (rainfall) modelling	Assessment of available regional and climate (rainfall) projections
Sensitivity	Mangrove condition	Literature review, and aerial and on ground image analysis
	Recruitment	
	Mortality	
	Seaward edge retreat	Aerial photograph and satellite image analysis of change by GIS
	Recent spatial changes of mangroves	
	Accretion rates under mangroves	Information from similar low island settings and local environmental data
	Adjacent ecosystem resilience	Literature review
Adaptive capacity	Mangrove protection status	Literature review
	Local management capacity	
	Stakeholder involvement	
	Elevations above the mangrove	

Results from component analysis were ranked following (Ellison 2015), with 1 indicating a low level of vulnerability, and 5 very high vulnerability (Table 2). Average vulnerability was calculated from the total of component rank scores divided by the number of components completed (Ellison 2015). The vulnerability assessment was conducted separately for the two mangrove types on the atoll, inland depressions and coastal habitats, to allow comparison.

**Table 2:** Ranking criteria of vulnerability assessment ( adapted from Ellison 2015)

Rank	1	2	3	4	5
Exposure components					
Relative sea level trends	Site uplifting	Site slightly uplifting	Site stable	Site slowly subsiding	Site rapidly subsiding
Tidal range	> 3 m	2–3 m	1.5–2.0 m	1–1.5 m	< 1 m
Sediment supply type	High	Fairly high	Medium	Fairly low	Low
Climate (rainfall) modelling	Becomes wetter	Rainfall unchanged	Somewhat drier	Moderately drier	Significantly drier
Sensitivity components					
Mangrove condition	No or slight impact	Moderate impact	Rather high impact	High impact	Severe impact
Recruitment	All species producing seedlings	Most species producing seedlings	Some species producing seedlings	Just a few seedlings	No seedlings
Mortality	< 4%	4–10%	10–20%	20–30%	> 30%
Seaward edge retreat	None or little	Some	Moderate	Significant	Very significant
Recent spatial changes of mangroves	None	Some	Moderate	Significant	Very significant
Elevation ranges of mangrove zones	> 60 cm	50–60 cm	30–50 cm	20–30 cm	< 20 cm
Accretion rates in mangroves	> 1 mm greater than RSLR	< 1 mm greater than RSLR	Equal to RSLR	< 1 mm less than RSLR	> 1 mm less than RSLR
Adjacent coral reef resilience	Very high	High	Moderate	Low	Very low
Adjacent seagrass resilience	Very high	High	Moderate	Low	Very low
Adaptive capacity components					
Mangrove protection legislation	Good	Fairly good	Moderate	Poor	None
Local management capacity	Good	Fairly good	Moderate	Poor	None
Stakeholder involvement	Good	Fairly good	Moderate	Poor	None
Elevations above mangroves	Migration areas very available	Migration areas mostly available	Some migration areas available	Few migration areas available	No migration areas available

Sea level trends were determined using data from the Permanent Service for Mean Sea Level (Holgate et al. 2013; PSMSL 2020), using monthly tide gauge data from the nearest tide gauges, at the capital Majuro (located 200 km to the north-east) and Kwajalein atolls (located 310 km to the north-west). Two tide gauge datasets exist for Majuro, Majuro B (1968–1999) and Majuro C (1993–2019) which were combined, and the longer dataset for Kwajalein (1946–2019) provided a longer sea level record. Linear regression was used to determine annual rates of change (mm year^−1^). Tidal range was calculated from the 2020 diurnal tidal predictions from the National Oceanic and Atmospheric Administration (NOAA) station Jaluit Atoll (SE Pass, Station ID: TPT2703) (NOAA 2020a). Tide maximums and minimums were averaged over the year to calculate the annual average tidal range.

Previous records of mangrove locations on Jaluit included some brief descriptions but no maps. Identification of mangrove areas used available aerial and satellite imagery, and methods for differentiation of mangroves included:

1. Analyzing literature to identify islands known on Jaluit where mangroves are present,
2. Identifying inland depressions with open water bodies (for inland mangroves),
3. Using experience from spatial change analysis of the mangrove atoll of Tarawa (Ellison et al. 2017) to identify mangrove-typical rounded tree crowns with even texture and dark coloration,
4. Lagoon shore mangroves showed colonizer juvenile trees growing offshore, these were used to confirm mangrove coloration and texture to verify more inland extents,
5. For lagoon coastal mangroves absence of beach and dry land between the vegetation and water line,
6. Creek passageways extending into forested areas (for lagoon coastal mangroves),
7. Evidence of mangrove tannins staining the water and sediments offshore of land areas.

Spatial change of mangrove areas over time were determined using ArcGIS Desktop (Version 10.8). Historical imagery (Table 3) was overlaid and georeferenced to a 2018–2019 base map using fixed structures such as buildings, slipway edges and rock outcrops, as found useful by other atoll spatial change studies (Ford 2013; Yates et al. 2013). In total, 132 historical images were obtained and georeferenced to the base map from 1945 (40), 1976 (44) and 2010–11 (48). This imagery informed the mangrove extent analysis and the shoreline accretion/retreat analysis. Polygons were created of the inland mangrove depressions using the methods for differentiation of mangroves listed above. Areas of open water and/or bare soil were removed from the total mangrove area calculation. The extent of inland mangroves for each aerial/satellite image were calculated using ArcGIS.

**Table 3:** Imagery used for Digital Shoreline Analysis System and mangroves spatial analysis

Available image date	Image	Time interval (years)	Cumulative time relative to base (years)	Clear status	Spatial resolution
1945	US National Archives (B&W aerials)	Base	Base	Clear	0.64 m
1976	United States Geological Survey (USGS) (B&W Aerials)	30	30	Very clear	0.21 m (1:8000)
2010/11	DigitalGlobe Image Mosaic (WV02)	44	74	Clear	0.60 m
2018/19^a^	Maxar Image Mosaic (WV02, WV03, WV04 & GE01)	8	82	Some cloud cover and low-resolution areas	0.31 m

^a^A small area of this Mosaic included a 2015 image covering the islands of Ae and Majrirok

Shoreline change was determined using the Digital Shoreline Analysis System (DSAS) version 5.0 add-in to Esri ArcGIS desktop (Himmelstoss et al. 2018). There were four areas identified with mangrove dominated shorelines, which were suitable for DSAS: Jaluit Jaluit, Aineman 1, Aineman 2, and Medyai. Two shoreline areas were not suitable for DSAS due to missing imagery. The DSAS on the Medyai shoreline was conducted only from 1976 through 2018/19 due to the lack of a mangrove shoreline present in 1945. All other shorelines had shoreline analysis conducted between 1945 through 2018/19. The DSAS settings were applied consistently to each shoreline analyzed with transect spacing length 5.0 m and a confidence interval of 90%. The default uncertainty of 10.0 m was applied to the analysis to account for error in georeferencing of historical imagery, human digitizing error and differences in the spatial resolution of the imagery used (Himmelstoss et al. 2018). The spatial resolution of aerial and satellite images used was less than 1.0 m pixel size (see Table 3), therefore an uncertainty of 1.0 m was adopted. Following the approach of Ruggiero et al. (2013) a standard error of 4.0 m for georeferencing and 1.0 m for digitizing error was also adopted. The square root of the sum of all squares was calculated for each of these uncertainty factors yielding a total error of 4.24 m (Ruggiero et al. 2013). A conservative approach was undertaken with the total uncertainty raised to 10.0 m, considering other possible sources of error such as vegetation shadow. The 10.0 m error applies to the shoreline uncertainty on actual shore placement, not the calculated rates which come from a best fit calculation (Himmelstoss et al. 2018).

Two rate-of-change statistics were calculated from the shoreline analysis: linear regression rate (LLR) and the end point rate (EPR). LLR is calculated by adding a linear regression line to each plotted shoreline point across a transect, while EPR is calculated by dividing the distance of the total shoreline change across the temporal change of the oldest and most recent measured shorelines (Himmelstoss et al. 2018).The EPR only requires two shoreline positions and as a result has a greater number of transects (Himmelstoss et al. 2018).

## Results

### Exposure components

**Fig. 2:**
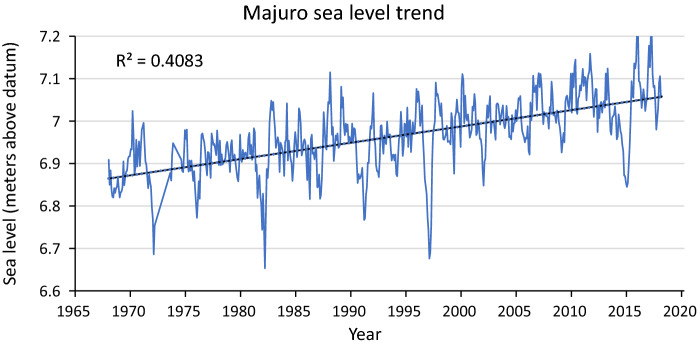
Sea level trend determined using data from the Permanent Service for Mean Sea Level (Holgate et al. 2013; PSMSL 2020) from Majuro Atoll Station C (combined with Majuro B) with linear regression line. Anomalies correspond with ENSO variability (Chowdhury et al. 2007)

Analysis of NOAA (2020a) data showed the average annual tidal range of Jaluit atoll to be 1.03 m, hence assigning a vulnerability rank (Table 2) of 4. These tide predictions are referenced to the Kwajalein tide gauge (Station ID: 1820000). Analysis of long-term tide gauge records from Majuro and Kwajalein (PSMSL 2020) showed rising relative sea level trends (Table 4). The Majuro record (Fig. 2) was used in the vulnerability ranking for this component, being located closer to Jaluit than Kwajalein. Evidence of increased sea level rise is also shown by satellite altimetry data, with 7 mm year^−1^ SLR indicated 1993–2011 (Australian Bureau of Meteorology and CSIRO 2011). The tide gauge information provides trends relative to the land more appropriate for mangrove vulnerability assessment, hence the rate calculated for Majuro justified a rank of 5 (Table 2) for the relative sea level trend component.

**Table 4:** Results of sea level trends from RMI

Tide gauge	Trend (mm year^−1^)	Time period
Majuro C	3.53 ± 1.8	1968–2019
Kwajalein	1.98 ± 0.65	1946–2020

A review of climate models from the Australian Bureau of Meteorology and CSIRO (2014) for RMI found that precipitation is expected to increase, with high confidence. The frequency of extreme rain events was also expected to increase, while droughts are expected to decrease in frequency (Australian Bureau of Meteorology and CSIRO 2014). This indicates low vulnerability for the climate (rainfall) modelling component. Thus, both the inland depression and coastal mangroves were assigned a rank of 1 (Table 2) for the climate modelling component.

The geomorphic setting of Jaluit atoll is a low-lying marine atoll which receives limited allochthonous sediment (Woodroffe 1992), resulting in a high vulnerability to RSLR of rank 5 (Table 2). Most sediments would be derived from biogenic carbonate sediments and from organic sediments derived from litter fall and root mat development within the mangroves (McKee 2007; Ward et al. 2016; Ellison 2019; Yamano et al. 2020).

### Sensitivity components

Mangrove forest health assessment was conducted using visual analysis of satellite images, site imagery obtained of a Jaluit Jaluit inland mangrove forest (one of the largest on the island), and evidence provided in literature. Some environmental investigations of the atoll occurred in the early 2000s prior to the Ramsar Site designation (Aiello 2001; Lindsay and Aiello 2003; Bungitak and Lindsay 2004). Overall, Jaluit Atoll’s mangrove forests have been reported to be in good health (Aiello 2001; Lindsay and Aiello 2003), with some impacts due to infrastructure and the overconsumption of some resources such as mangrove crabs (Lindsay and Aiello 2003). Spatial analysis indicated human impacts in some locations (Fig. S2). Satellite imagery indicated close to 100% canopy coverage in most mangrove areas, with no indication of dead trees present. Similarly, spatial analysis showed areas of robust offshore recruitment of new mangrove saplings and the presence of new colonisers (likely *Rhizophora*), supporting a low vulnerability rank of 1 (Table 2) for recent spatial changes of mangroves.

**Fig. 3:**
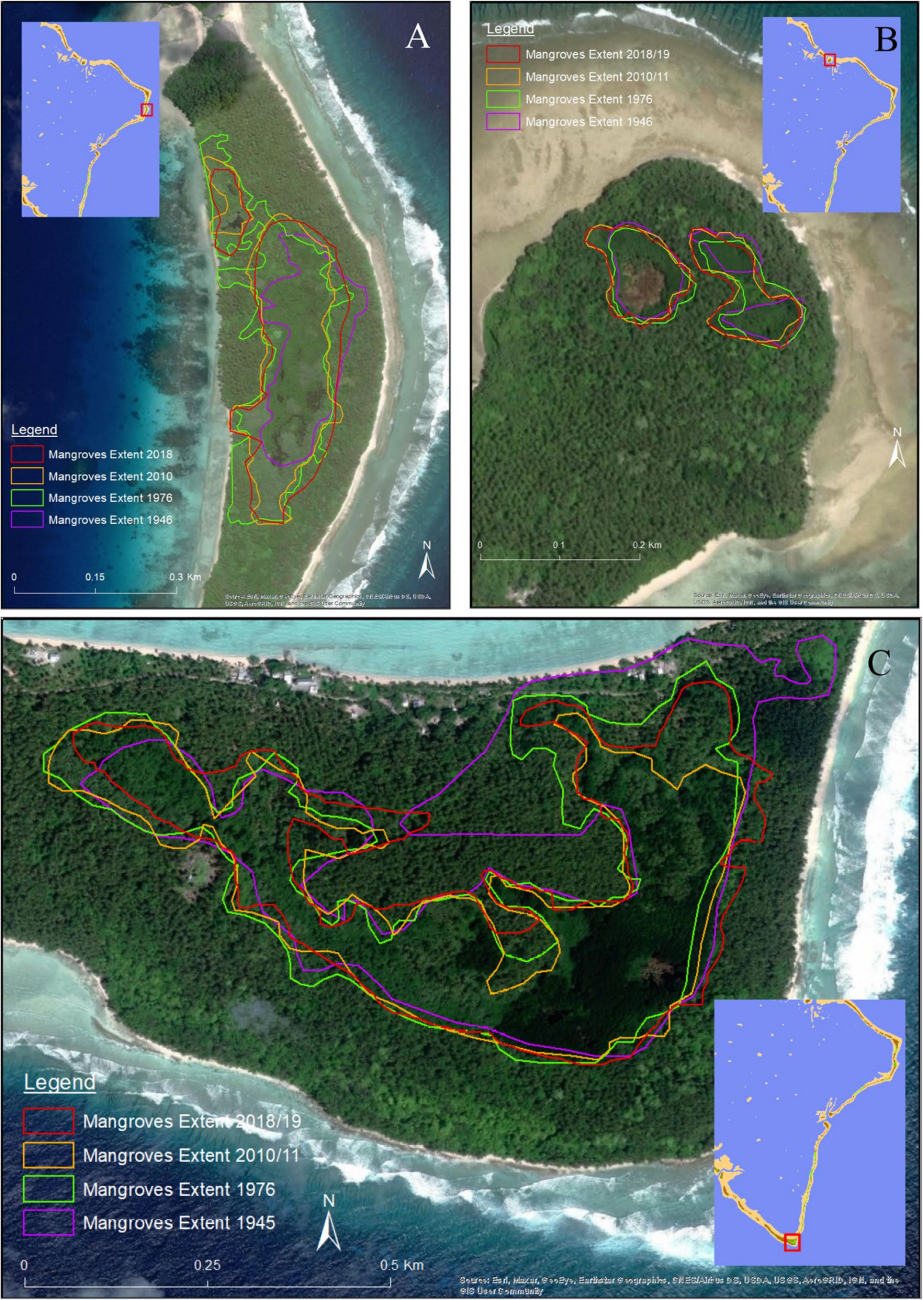
Spatial change of inland mangroves on Jaluit islands **A.** Imiej, **B.** Kinajon 1 (left) and 2 (right), **C.** Jaluit Jaluit 1

The mangrove area changes showed an overall increase in the area of the inland mangroves (Table 5), with results for individual islands shown in Table S1. There was a reduction between 1945 and 1976, before an increase by 2010/11, and continuing increase through to 2018/19. Examples of temporal changes in inland mangrove forests are shown in Fig. 3, Fig. S1. Results therefore indicate the lowest vulnerability rank of 1 for the components of seaward edge retreat and recent spatial changes (Table 2).

**Table 5:** Spatial changes in area of Jaluit inland mangroves

Year	1945	1976	2010–2011	2018–2019
Total area hectares (ha)	42.9	40.1	42.8	49.8

**Fig. 4:**
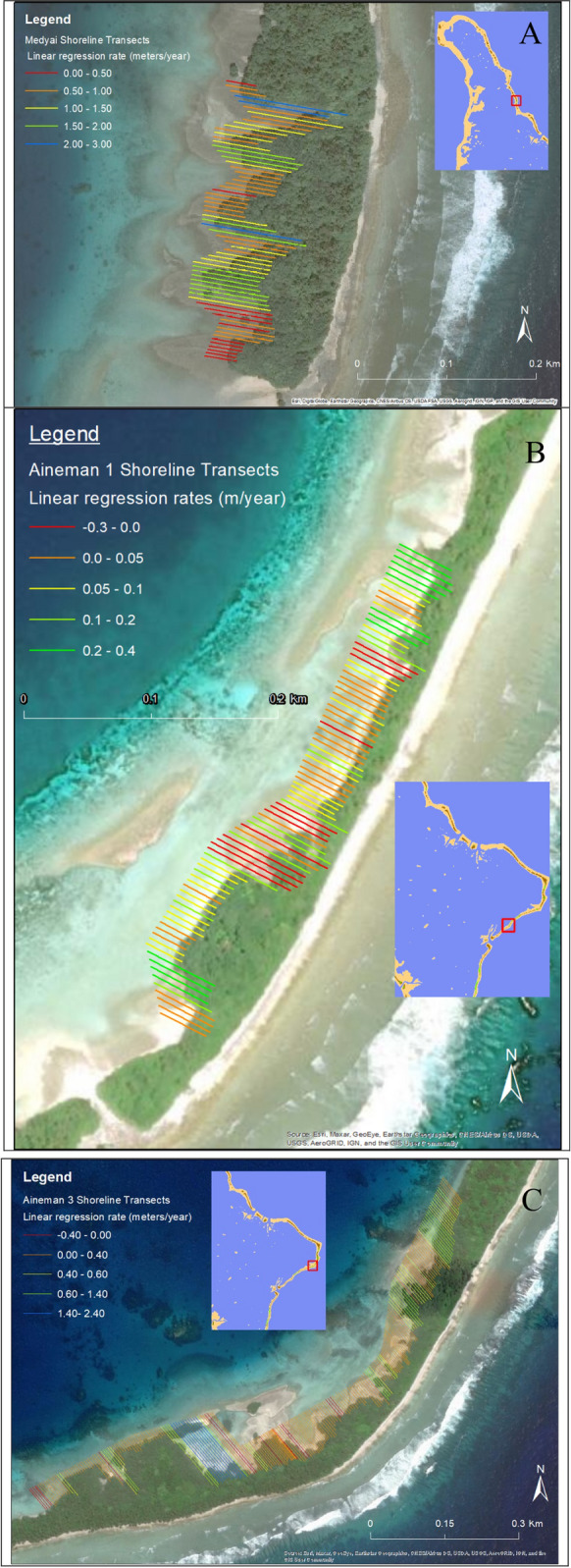
Lagoon mangrove DSAS results **A.** Medyai, **B.** Aineman 1, **C.** Aineman 2

Mangrove seaward edges showed progradation along five of the six identified areas with mangrove dominated shorelines (Table 6; Fig. 4, S10–S20). Ertok (the smallest shoreline area) rather became denser in vegetation coverage with slight reduction in area from 1.56 to 1.48 ha between 1945 and 2018/19. Two sections of Aineman were analyzed, while a third shoreline on Aineman lacked suitable imagery for the DSAS but showed increase from virtually no mangroves in 1976 to a robust mangrove shoreline by 2010. Progradation was predominant along the largest mangrove shores, with LRRs indicating progradation at Medyai, Aineman 1, Aineman 2 and Jaluit Jaluit in 100%, 88.5%, 94.6% and 95.7% of all transects respectively, with rates of increase of 1.07, 0.1, 0.38 and 0.51 m year^−1^. The lowest rank of vulnerability of 1 was therefore assigned (Table 2), given strong mangrove progradation across the atoll.

**Table 6:** DSAS results from the lagoon margin mangrove areas of different islands on Jaluit atoll

Mangrove area	Medyai	Aineman 1	Aineman 2	Jaluit Jaluit
Total number of transects	65	95	243	2,519
Measured shoreline length (km)	0.32	0.47	1.21	12.59
EPR (End Point Rate)				
Erosional transects (% of total)	0	22 (23.2%)	29 (11.9%)	138 (5.5%)
Average of all erosional rates (m year^−1^)	N/A	− 0.18	− 0.11	− 0.18
Progradation transects (% of total)	65 (100%)	73 (76.8%)	214 (88.1%)	2,318 (94.5%)
Average of all progradation rates (m year^−1^)	1.02	0.11	0.35	0.47
LLR ( Linear Regression Rate)				
Erosional transects (% of total)	0	10 (11.5%)	13 (5.4%)	98 (4.3%)
Average of all erosional rates (m year^−1^)	N/A	− 0.1	− 0.08	− 0.16
Progradation transects (% of total)	65 (100%)	77 (88.5%)	229 (94.6%)	2,174 (95.7%)
Average of all progradation rates (m year^−1^)	1.07	0.1	0.38	0.51

Review found similar environmental conditions to Jaluit Atoll on the barrier reef islands of Belize, located offshore with no input of allochthonous sediments rather formed on a limestone platform through biogenic processes (McKee et al. 2007; McKee 2011). Fringing mangrove settings represent the most similar setting to Jaluit coastal mangroves, where surface elevation table (SET) results showed elevation change of + 4.1 mm year^−1^, primarily from organic sources (McKee et al. 2007). Long-term tide gauges on Majuro (Fig. 2) 1968–2018 indicated rising RSL trends of 3.53 ± 1.8 mm year^−1^, and relative to the net accretion rate of 4.1 mm year^−1^ from fringing mangroves in Belize (McKee et al. 2007) indicates a vulnerability rank of 2. Core results from inland mangroves on Funafuti Atoll, Tuvalu, showed a thin mangrove peat with vertical accumulation rates of 0.4–3.9 mm year^−1^ (Yamano et al. 2020), however net accretion rates of inland mangrove depressions have not been studied using SETs, and this question remains a gap in knowledge that future research could investigate.

Coral reef ecosystems of Jaluit, based upon studies and reports undertaken of coral health, indicate that health and resilience is high relative to other parts of the RMI and coral reefs globally (Beger et al. 2008; Baker et al. 2011; SPREP 2016; Thaman 2018). Limited threats to coral reefs include some coral bleaching, overharvesting of reef invertebrates and herbivores, eutrophication and illegal fishing activities (Lindsay and Aiello 2003; Beger et al. 2008), indicating a low vulnerability ranking of 2 (Table 2). The increasing threat of warmer oceans causing more bleaching events and more intense tropical cyclones (Australian Bureau of Meteorology and CSIRO 2014) precludes the lowest vulnerability of 1.

A single species of seagrass occurs on Jaluit, *Thalassia hemprichii* (Tsuda et al. 1977; Tsuda and Sukhraj 2016). There is no documented evidence of exploitation of seagrasses on Jaluit, though a single species is more vulnerable to climate change than more diverse communities (Mori et al. 2013; Sakschewski et al. 2016). Seagrasses in the tropical Pacific are at risk from climate change (Waycott et al. 2011), however increased CO_2_ availability may benefit seagrasses, particularly *Thalassia hemprichii* (Jiang et al. 2010). The net impacts of climate change are expected to be negative on tropical Pacific seagrasses, with losses of 5–20% likely by 2035 in moderate IPCC scenarios (Waycott et al. 2011). Overall, reviewed evidence justifies a moderate vulnerability ranking of 3 (Table 2).

### Adaptive capacity components

There are strong traditional management processes and willingness among local people to protect their environments, as evidenced by the traditional ‘Mo’ taboo or no-take areas (Baker et al. 2011). However, the RMI is a poor country with few income sources; Jaluit has annual household incomes of < 2000 (USD) as stated in the most recent census (Economic Policy Planning and Statistics Office and SPC 2011). Jaluit is a very large atoll (Fig. 1) with a low and dispersed population across isolated areas, making protection and management of the environment difficult (Lindsay and Aiello 2003). Therefore, the capacity and willingness to protect local environmental assets such as mangroves may be outweighed by the necessity for locals to maintain their livelihoods. This is indicated by some residential beach/aggregate mining on Jaluit (Lindsay and Aiello 2003).

Robust laws around the protection of mangroves and coastlines prevail on Jaluit, with several pieces of legislation complimenting traditional conservation measures such as the ‘Mo’ system (Baker et al. 2011). Jaluit atoll has recognised conservation status zones including 14 areas of ‘no-take’ and seven of ‘subsistence-only’ (SPREP 2016). However, according to the State of the Environment Report in 2016, national enforcement of these laws is inconsistent in places and poor in others (SPREP 2016). The lack of resources to monitor the environment and absence of enforcement remains an issue for mangrove protection status, indicating a ranking of 3 (Table 2) for mangrove protection status and local management capacity.

Stakeholder engagement in conservation management processes is strong within the RMI with the Reimaanlok system, recognized internationally as a progressive coastal zone management approach particularly among SIDS (Sterling et al. 2017). The establishment of this national conservation framework seeks the engagement of traditional landowners and chiefs, ‘Iroij’, and revives the traditional ‘Mo’ concept (Baker et al. 2011; Office of Environmental Planning Policy Coordination 2017). Western style concepts of protected areas such as ‘no take’ zones are adapted to the local context, where conserved areas are more sophisticated, empowering local stakeholders (Baker et al. 2011). During the creation of the Jaluit Atoll Conservation Area, seven community meetings occurred and community members raised concerns about commercial harvesting, poor enforcement of traditional and modern laws, and lack of knowledge on reef biology (Lindsay and Aiello 2003). Cooperation in these meetings was very high, with all accepting stricter management of resources, and extending formal support to the conservation plan (Lindsay and Aiello 2003). These examples indicate good levels of stakeholder engagement in conservation on Jaluit, justifying a low vulnerability rank of 1 (Table 2) for this component.

Areas for inland migration of mangroves was ranked with a high vulnerability of 4 (Table 2), because all of Jaluit is below 2 m (Deunert et al. 1999) and islands are limited in spatial extent (Fig. 1). Results from the Jaluit mangrove vulnerability assessment are compiled in Table 7.

**Table 7:** Vulnerability assessment rank results for Jaluit mangroves

Components	Inland mangrove depressions	Coastal
Exposure		
Relative sea level trends	5 (rapid SLR)	5 (rapid SLR)
Tidal range	4 (1–1.5 m)	4 (1–1.5 m)
Sediment supply type	5 (low)	5 (low)
Climate (rainfall) modelling	1 (becomes wetter)	1 (becomes wetter)
Sensitivity		
Mangrove condition	2 (moderate impact)	2 (moderate impact)
Recruitment	3 (some species producing seedlings)	N/D
Mortality	1 (< 4%)	1 (< 4%)
Seaward edge retreat	1 (mangrove aquatic margins prograding)	1 (none or progradation)
Recent spatial changes of mangroves	1 (none or expanding)	1 (progradation)
Accretion rates under mangroves	N/D	2 (> 1 mm greater than RSLR)
Adjacent coral reef resilience	2 (high)	2 (high)
Adjacent seagrass resilience	3 (moderate)	3 (moderate)
Adaptive capacity		
Mangrove protection status	3 (moderate)	3 (moderate)
Local management capacity	3 (moderate)	3 (moderate)
Stakeholder involvement	1 (good)	1 (good)
Elevations above mangroves	4 (few migration areas available)	4 (few migration areas available)
Average vulnerability	2.6	2.53

The assessment ranking results indicated a similar average vulnerability for both inland mangrove depressions (2.6) and lagoon shoreline mangroves (2.53). Both mangrove types indicated similar components of vulnerability and resilience as they are subject to the same exposure and adaptive capacity components.

## Discussion

### Specific areas of vulnerability

Over the twenty-first century, RSLR and the lack of higher elevation migration areas will become a vulnerability for the mangroves on Jaluit, similar to settings on other atoll islands (Oppenheimer et al. 2019; Constance et al. 2021). The low tidal range (Table 7) adds vulnerability, as mangrove sediment soil redox conditions and a sulfate reduction realm (Ward and de Lacerda 2021) bring risks for successful relocation onto what is currently dry land. Tide gauge records from the RMI also indicate recent higher rates of RSLR of 3.53 ± 1.8 mm year^−1^ relative to global trends (Table 4), causing a high vulnerability rank for RSLR. Results allow an update or correction (Table 7) to Ellison’s (2015) categorisation, that high RSLR must indicate subsidence, whereas it could also result from ENSO variability (Chowdhury et al. 2007). Mangrove areas in this study did not show negative impacts from the recent RSLR, although ongoing monitoring is of great importance for atolls at risk from SLR (Ford and Kench 2016).

Interior mangrove forests benefit from increased tidal flushing (Lindsay and Aiello 2003), therefore they may have greater resilience with possible increases in suitable flooded habitats with SLR. As sea level rises and flood events become more frequent, inland mangrove depressions may in the short to medium term be better placed than terrestrial forest to benefit from these changes across the atoll, a phenomenon observed along the Northern Gulf of Mexico (Doyle et al. 2010). In the longer term, with increased SLR, the overall migration areas available will likely be reduced (Ward et al. 2016) resulting in an increasing vulnerability.

Low-lying island carbonate settings lack the catchment derived minerogenic sediment supplies that benefit mangroves of high islands and continental landmasses (McIvor et al. 2013). Reef islands and atolls rely solely on autochthonous sediments derived from atoll biotic productivity, including mangrove organic matter of litter fall and root mat growth (Cahoon et al. 2003; McKee et al. 2007; McIvor et al. 2013). These settings share a similar vulnerability in that accretion is dependent on healthy mangrove productivity. Sedimentation rates may be enhanced in coastal areas by increasing mangrove root networks, and the carbonate sediment production provided by coral reefs and seagrass areas (Perry et al. 2015). Coral reefs and seagrasses are primary sites of sediment production on atolls such as Jaluit, and associates like parrotfish (Scaridae) enhance the sediment supply through bioerosion (Woodroffe 1992; Kench and Cowell 2002; Horstman et al. 2014; Perry et al. 2015). Atoll lagoon mangrove margins in Kiribati showed sediment derived from corals, molluscs, foraminifera and calcareous algae (Ellison et al. 2019), indicating the importance of adjacent reefs and seagrasses to mangrove sediment supply. The sedimentation sources of Jaluit mangrove shorelines and interior sites both require future on-ground assessment.

For mangrove protection status and local management capacity, vulnerability was lower (Table 7) and could be further reduced through capacity building (Ellison 2015). There are strong protection laws and documented willingness among Jaluit locals to sustainably manage the environment, however, both resources and capacity are lacking, a problem occurring across many Pacific Island countries (SPREP and EDO NSW 2018). Limited enforcement and a lack of resources to police the protection legislation is noted (Lindsay and Aiello 2003; Jupiter 2014). Improving management is important in reducing the vulnerability of mangroves and the wider ecosystems, and is achievable by well-funded projects (Ellison 2012). Community awareness and education programs which emphasize the importance of mangroves in disaster risk reduction, erosion control and as nurseries for fish and other species are cost effective methods to empower local people and increase environmental stewardship (Jupiter 2014). Similarly, citizen monitoring of environmental compliance allows empowerment of island communities and reduces unsustainable harvesting of resources (Jupiter 2014). Sustainable sources of funding for conservation, improved data collection and reporting can enhance resilience (Jupiter 2014; Keppel et al. 2014).

Exposure components including tidal range, RSLR, sediment supply type and climate (rainfall) changes are consequent from site position, climate and geography. These cannot be directly managed (Ellison 2015), though adaptation actions can mitigate risks, and managers can effectively focus on improving the components of sensitivity and adaptability. Similar mangrove vulnerability occurs in Douala Estuary (Cameroon), Tikina Wai (Fiji), and Yucatan Peninsula (Mexico), with low tidal ranges combined with limited sediment supply rates or subsidence (Ellison 2012, 2015; Ellison and Zouh 2012; Cinco-Castro and Herrera-Silveira 2020). Sites with resilience to exposure components include Rufiji Delta (Tanzania) and Guangxi (China) with larger tidal ranges and higher rates of sediment supply due to the presence of large rivers (Ellison 2012; Li et al. 2015).

### Specific areas of resilience

Increased temperatures are expected to impact mangroves at primarily higher latitudes (Osland et al. 2017, 2020), with little impact expected at equatorial latitudes where Jaluit is located (Waycott et al. 2011). However, changes in freshwater availability will be relevant, with mangroves benefitting from increased freshwater (Krauss et al. 2007). Results from review of climate models indicated that total rainfall and intensity are likely to increase in the RMIs over the 21st century (Australian Bureau of Meteorology and CSIRO 2014). Although mangroves are saltwater tolerant, increased availability of freshwater increases productivity, and has been positively correlated with both productivity and tree growth (Krauss et al. 2007; Ward et al. 2016). Mangroves of Jaluit and elsewhere in the RMI will benefit from these projected changes in rainfall, a resilience gained relative to low carbonate mangrove settings at higher latitudes which may experience rainfall reduction as well as impacts from hurricanes (Cahoon et al. 2003).

The resilience of adjacent coral reef ecosystems to climate change, and their overall health, is vital to mangroves as coral reefs provide sediment upon which mangroves can accrete (McLeod and Salm 2006). Atolls form on coral platforms, therefore depend on coral reefs for geological formation (Grigg 1982). Reefs also buffer from persistent wave action and storm events, and provide habitat for foraminifera which also contribute sediment supply (McLeod and Salm 2006; Beger et al. 2008; Ellison et al. 2019). The threat of coral bleaching events with warmer ocean temperatures is expected to become more likely with climate change, a process which is difficult to mitigate (Sully et al. 2019). However, the protection of ‘key-stone’ species related to coral reef health in conjunction with improved management of nutrient and sediment runoff could assist (McCook et al. 2009). Continued protection of coral reefs is vital to the health of the mangroves owing to ecosystem service-related benefits to mangroves (Moberg and Folke 1999).

Stakeholder involvement across Jaluit is a component of inherent resilience underpinned by the renowned ‘Reimaanlok’ the RMI national framework for conservation and resources management (Baker et al. 2011). Continued usage of this framework engaging landowners, chiefs, community and local governments will ensure a high level of stakeholder contribution to mangrove resilience.

Mangrove health, seaward edge retreat and reduction in mangrove area are three further components which received the lowest vulnerability ranking (1) and are discussed in the spatial analysis section below. Like Tikina Wai (Fiji), Jaluit has areas of resilience in mangrove health, coral reef resilience and stakeholder involvement (Ellison 2012, 2015). The overall vulnerability was lower for the mangroves of Tikina Wai (1.9) partly due to geomorphic setting (high island), but also on-ground measurements of mangrove basal area, basal area change and litter productivity, all of which showed high resilience (Ellison 2012, 2015). Owing to COVID-19, this study was unable to include fieldwork, focussing on remote sensing and literature review methods that enabled the vulnerability assessment to be conducted rapidly and at low cost. This is a useful example because there are many remote mangrove areas including atolls and they are very vulnerable to SLR, but it is difficult and expensive to conduct on the ground studies in such remote locations.

### Spatial changes of mangrove areas

Inland basin mangroves were found on eight of Jaluit’s islands including Ae, Emidj, Ewo, Imiej, Jaluit Jaluit, Kinajon, Mejrirok and Pinglep (Fig. 3, S3–S8). Results showed inland mangrove areas decreased between 1945 and 1976 (Table 5), consistent with the results of Ford and Kench (2016) finding contemporaneous decrease in the entire atoll island mass, due to the erosional impacts of Typhoon Ophelia in 1958 (Blumenstock 1958). Following 1976, results showed the overall area of inland mangroves expanded (Table 5), while two inland forests reduced area on Jaluit Jaluit Island (Table S1). Over time, several mangrove spatial change patterns were observed including:

1. lagoonal closure, where mangrove colonizers eventually close off areas of lagoon and reef flats,
2. tidal creek infill, where creeks from inland mangroves become closed off from the lagoon, and
3. forest transition, where mangrove forest gives way to littoral forest, such as coastal accretion blocking connection with tidal waters.

Observed overall increase in area (Table 5) and these patterns of change show adaptive resilience of inland mangrove forests. High resilience was also identified from seaward edge shoreline change analysis (Table 6), with prograding mangroves along each of the main mangrove shores (Figs. 4, S9–S20). The shoreline uncertainty value used in the DSAS (the default 10.0 m) is integrated into the calculation of standard error, confidence interval and correlation coefficient for the output statistic LRR (Himmelstoss et al. 2018). The 10.0 m error applies to the shoreline uncertainty on actual shore placement, not the calculated rates (Table 6) which come from a best fit calculation (Himmelstoss et al. 2018). One of these shorelines in 1945 did not show a mangrove shoreline, which may suggest island changes in 1976 following a Typhoon Ophelia. Shoreline advances also contributed to the process of mangrove transition towards littoral forest and the overall island building process. The demonstrated shoreline progradation is a sign of resilience and is in contrast to the global trends of mangrove loss (Giri et al. 2011). However, ongoing monitoring is required of the mangrove shorelines as there may be a tipping point at which the mangroves can no longer keep pace with rising sea levels (Eslami-Andergoli et al. 2015).

## Conclusions

This study has added an atoll perspective to understanding of future climate change vulnerability of mangroves, with Pacific low islands identified as having specific deficiencies in study (Kuruppu and Willie 2015). Despite the exposed geomorphic setting and high rates of relative sea level rise, spatial evidence of strong mangrove progradation indicates unexpected resilience. However, this may not be the case if SLR increases over the current century, the IPCC (2021) articulating high confidence that sea levels will very likely continue to rise, storm surges and waves will exacerbate coastal inundation, and will cause small islands coastlines to retreat. This study provides a practical example of how vulnerability assessment of mangrove areas (Ellison 2015) can be conducted even using remote sensing and review techniques. On ground research on Jaluit, when Pacific travel becomes possible post COVID-19, could focus on mangrove community structure and basal areas, net accretion rates (McKee 2011) and elevations within the mangroves (Ellison et al. 2022), and study of the unique inland depression mangrove systems. Vulnerability assessment research could use such data to directly compare atolls with carbonate settings of the Caribbean and Central America, to gain information on mangrove resilience building. Future mangrove vulnerability assessments for Pacific Island atolls should incorporate local knowledge of ecological and socio-economic conditions whenever possible.

The mangroves of Jaluit Atoll are spatially small relative to those of high islands and continents, however, they are critical to island biodiversity, coastal protection, and co-benefits with adjacent reef environments such as providing nurseries for juvenile marine fauna including threatened species. The combination of wetlands of coral reef, lagoon, seagrass, coastal mangrove and inland mangroves in the Ramsar designation indicates the integral co-existence of these systems in making the atoll a unique place.

## Supplementary Information

Below is the link to the electronic supplementary material.

Supplementary file1 (PDF 4082 kb)

## Data Availability

The datasets generated during and/or analysed during the current study are available in the supplementary information. All spatial analysis results of inland mangrove forests along with figures indicating temporal mangrove extents can be found in the supplementary information.
