# Supplementary material for: Atoll inland and coastal mangrove climate change vulnerability assessment
Source: Wetl Ecol Manag. 2022 May 11;30(3):527–46. doi: 10.1007/s11273-022-09878-0 (PMC9091136; doi:10.1007/s11273-022-09878-0)
Supplement: Supplementary file 1 — Supplementary file1 (PDF 4082 kb) [file 11273_2022_9878_MOESM1_ESM.pdf]

Crameri and Ellison 2022 Supplementary Material

Nicholas J. Crameri, Joanna C. Ellison

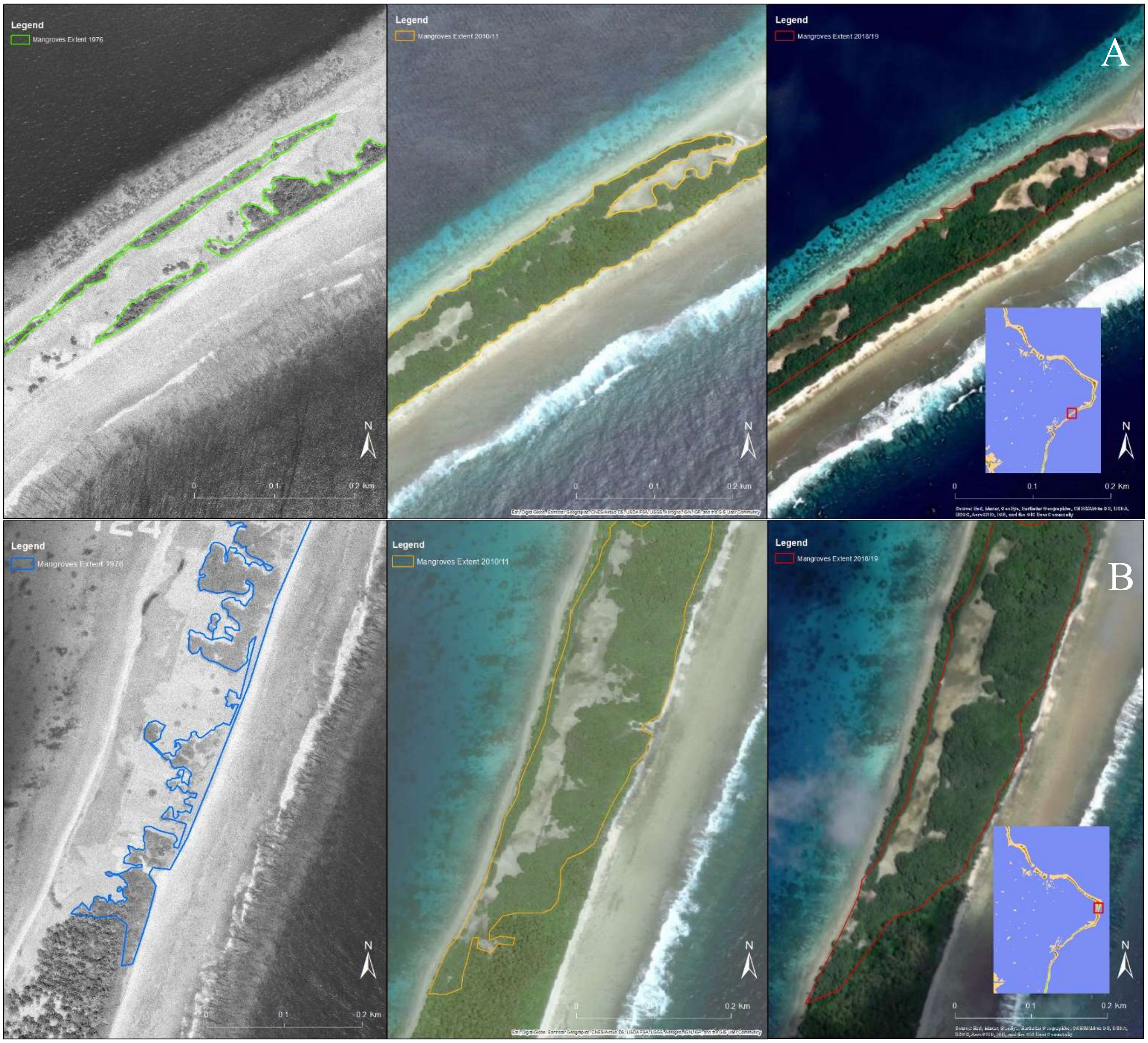

Fig. S1 Spatial changes of Jaluit mangroves. A. Aineman Island showing on the left a 1976 image indicates coastal mangrove areas, the middle 2010-11 image indicates the mangrove areas becoming denser and closing off a small section of the lagoon flats, the image on the right indicates mangroves becoming denser in 2018/19. B. Emidj Island, left 1976 image indicates coastal mangrove areas, the middle 2010-11 image indicates the mangrove areas closing off from the lagoon flats and transitioning to inland mangroves, the image on the right indicates mangroves becoming denser and further transitioning to inland mangroves in 2018/19.

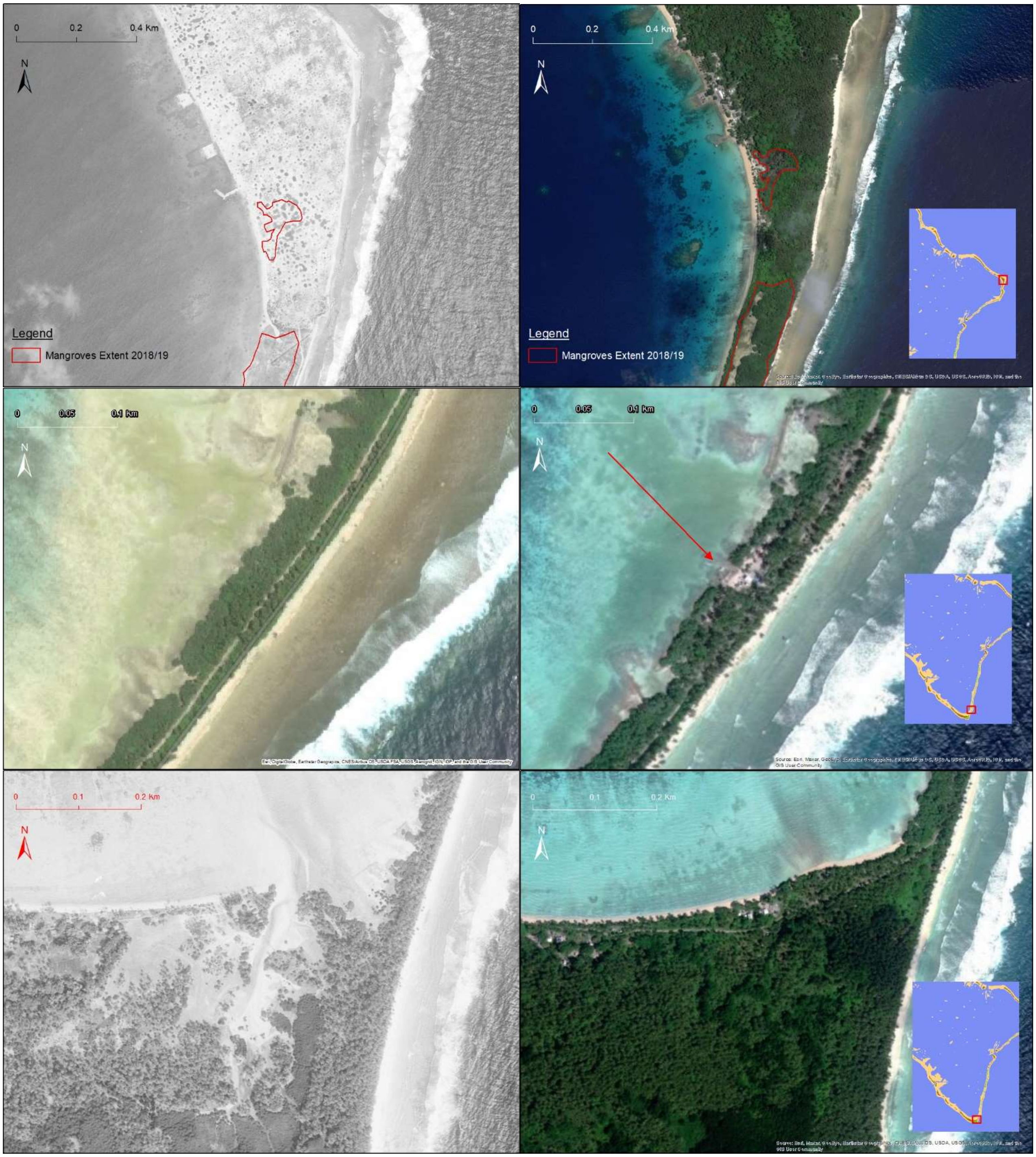

Fig S2 Human impacts on mangroves. A. On the left 1945 image indicates craters caused by WWII Allied bombing with the overlay of 2018/19 mangrove areas, on the right is the 2018/19 image of the same location. B. On the left is the 2010/11 mangrove shoreline on Jaluit, the right displays the same location in 2018/19 with clearance of mangrove habitat indicated by the red arrow. C. The image of the left displays 1945 Jaluit Jaluit inland mangroves with a small tidal creek running inland, the right displays the same location in 2018/19 with the creek infilled by a coastal road.

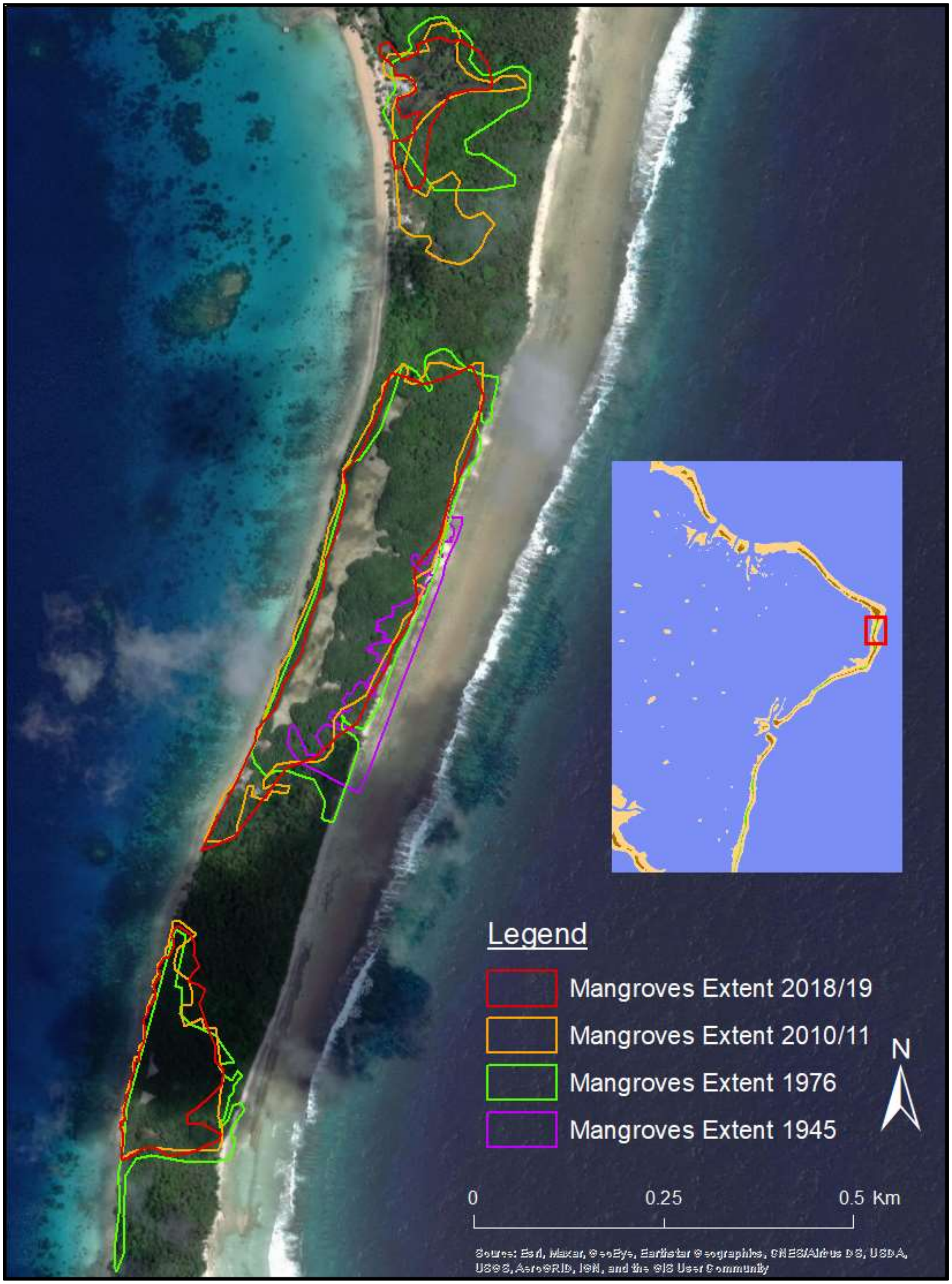

Fig S3 Inland Mangroves Emidj 1 (top), 2 (middle) and 3 (base)

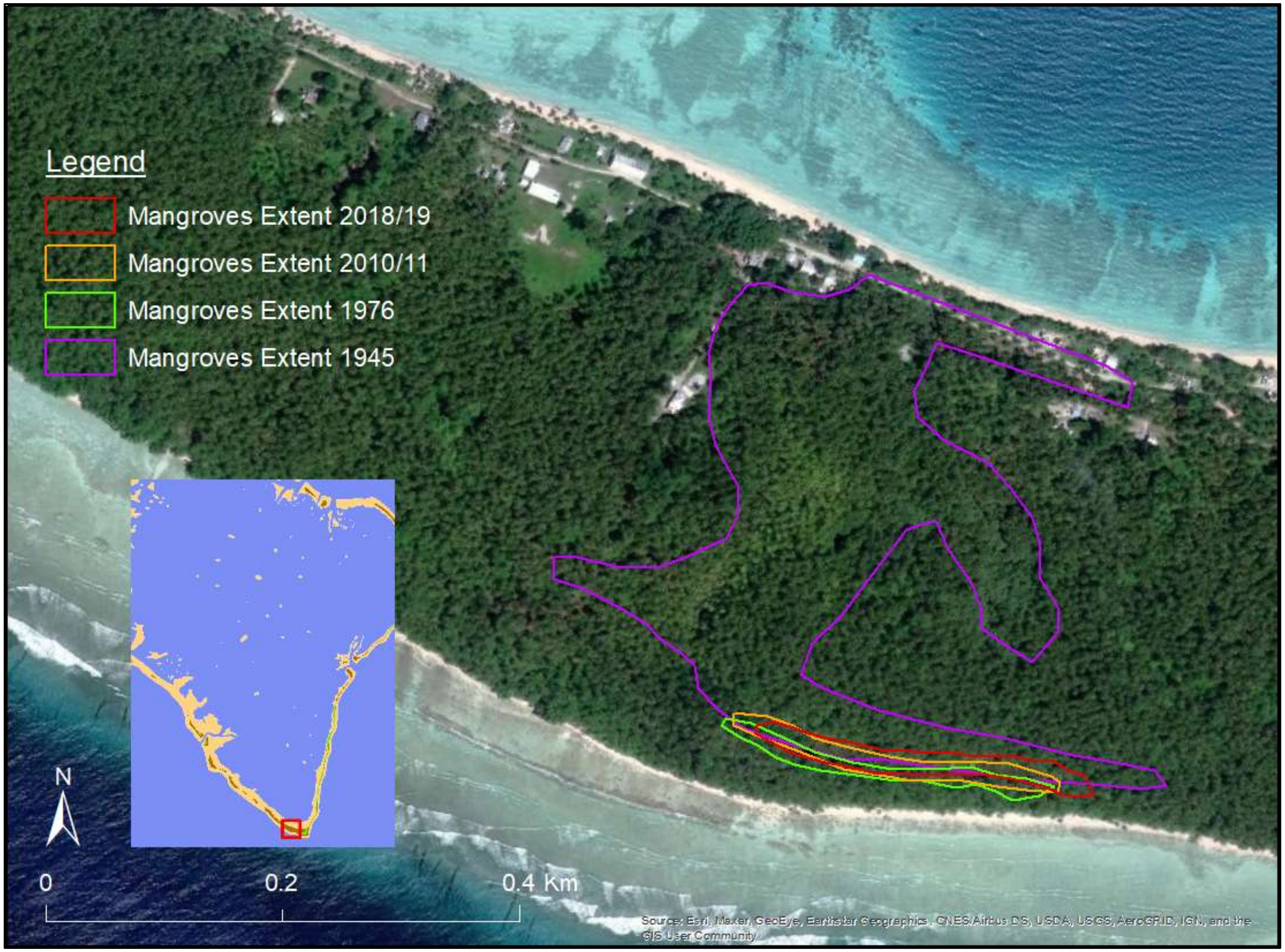

Fig S4 Inland Mangroves Jaluit Jaluit 2

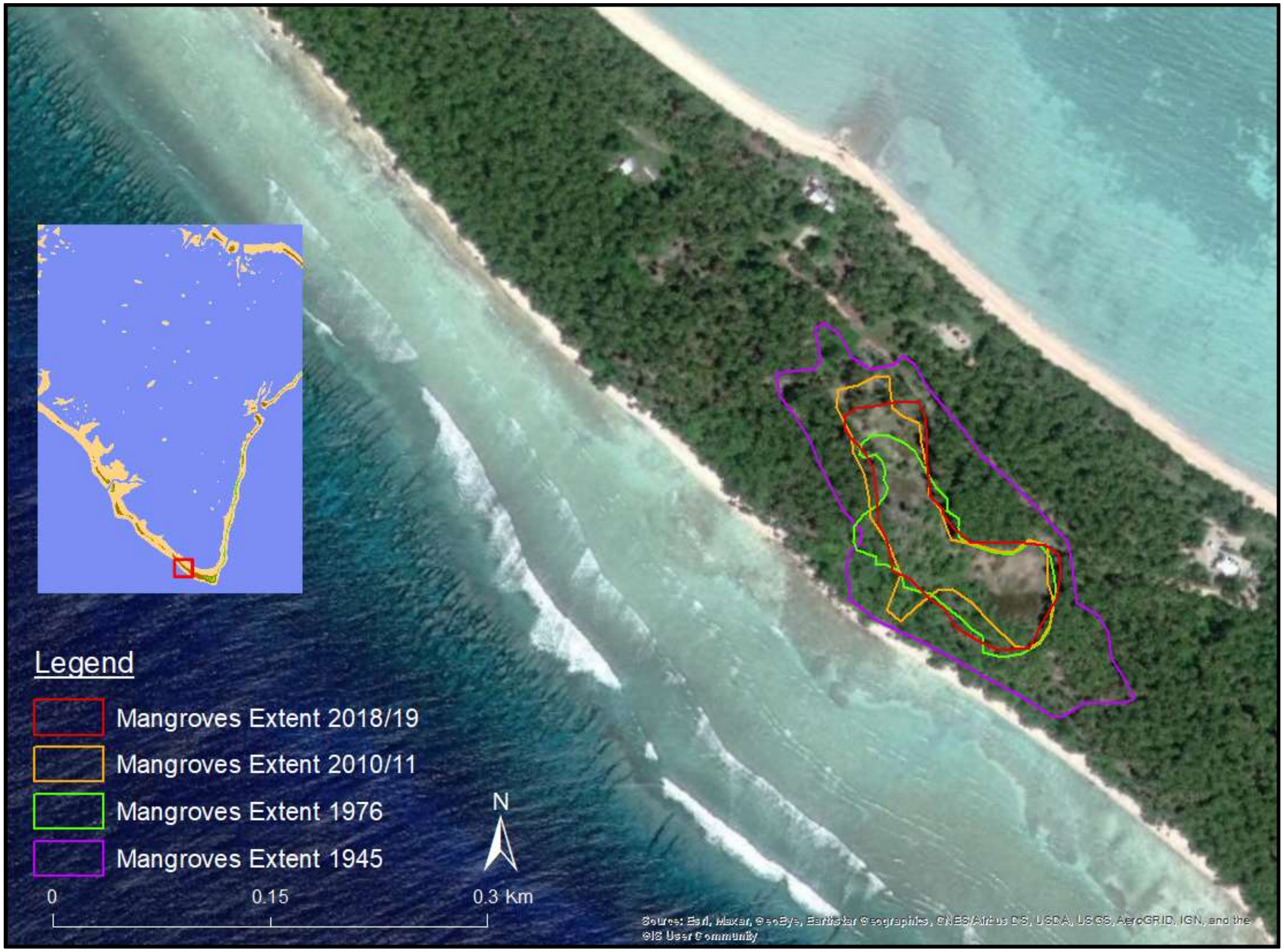

Fig S5 Inland Mangroves Jaluit Jaluit 3

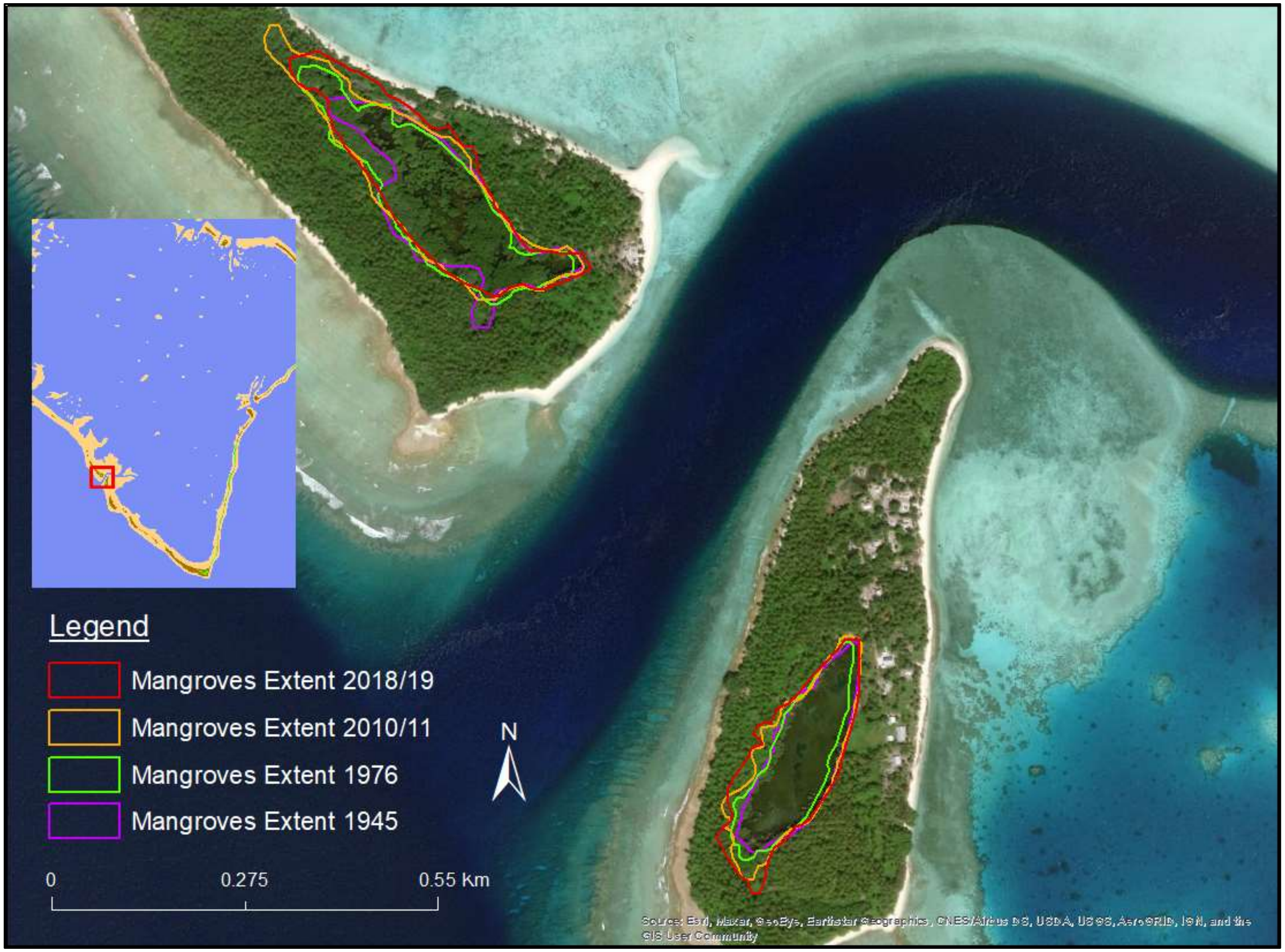

Fig S6 Inland Mangroves Ae (left) and Mejrirok (right)

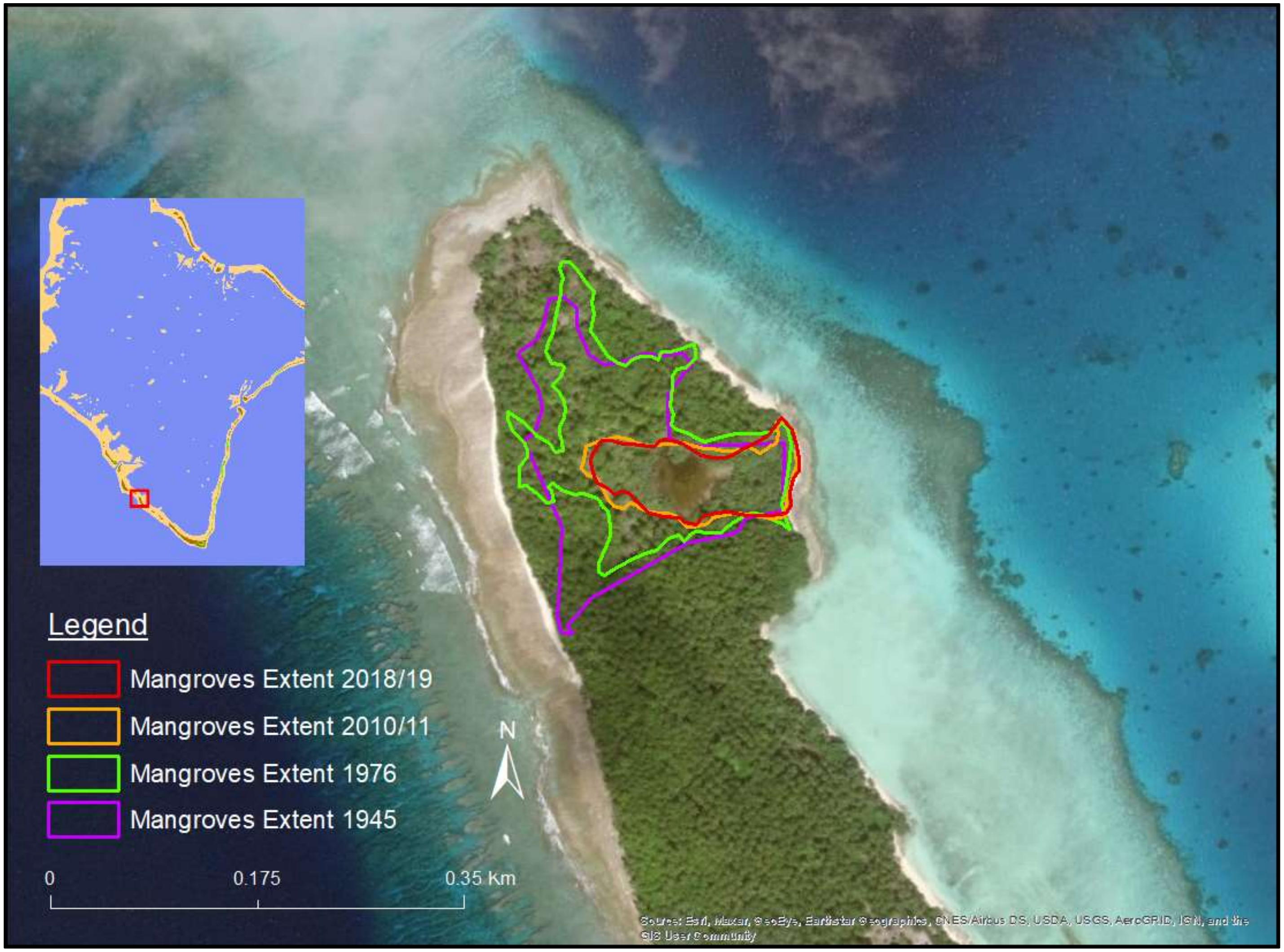

Fig S7 Inland Mangroves Ewo

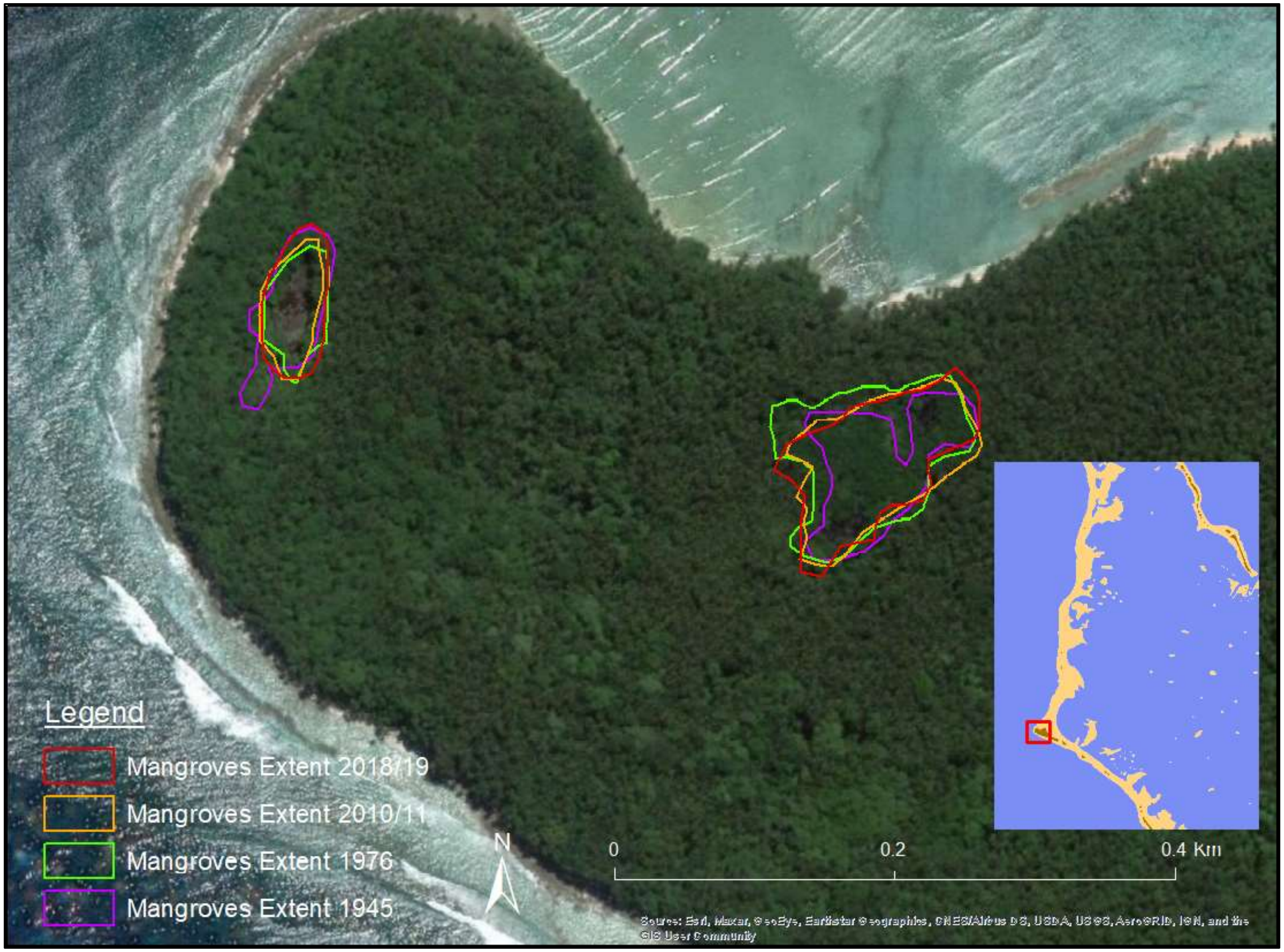

Fig S8 Inland Mangroves Pinglep 1 (left) and 2 (right)

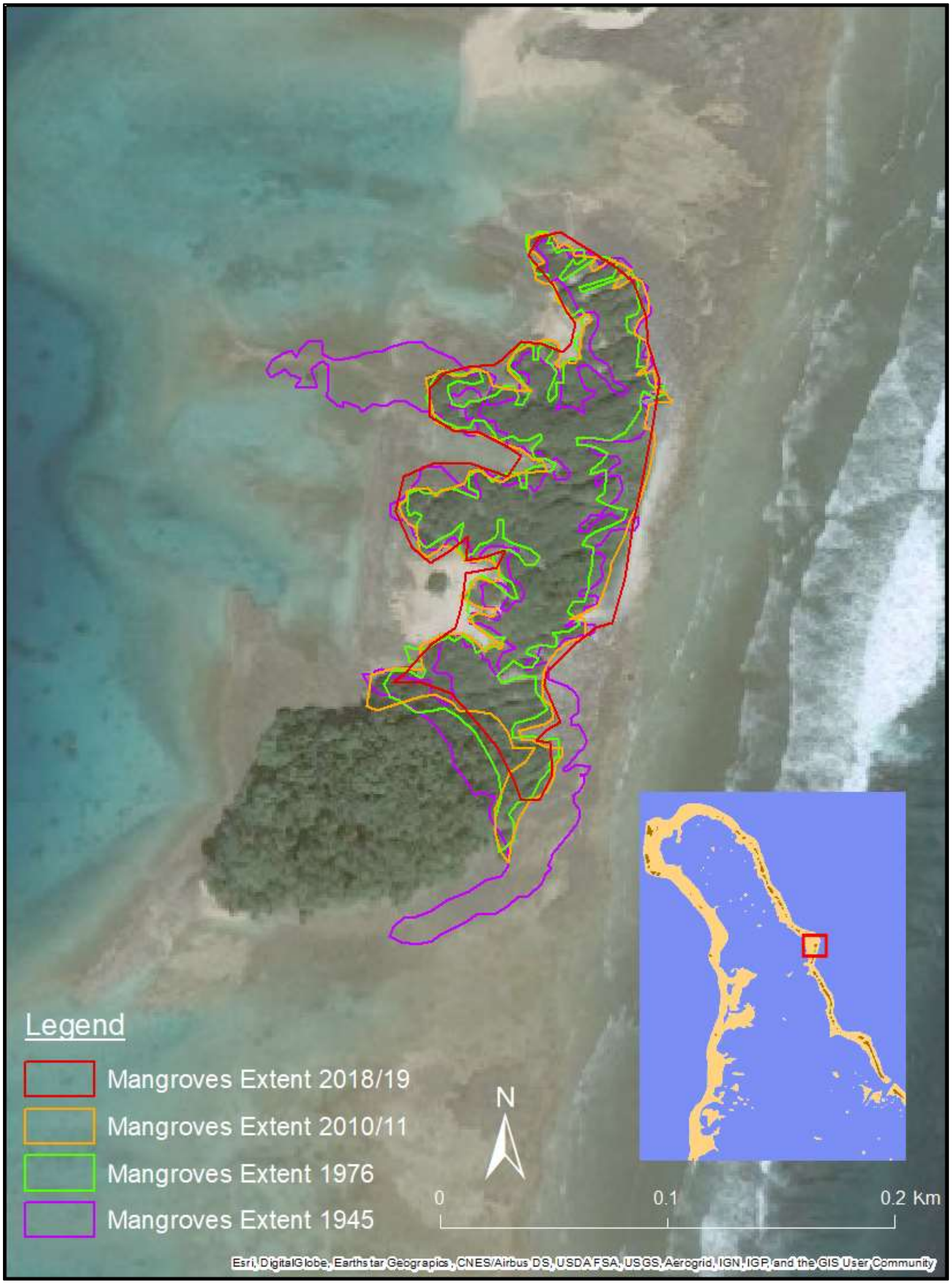

Fig S9 Mangrove shoreline change, Ertok

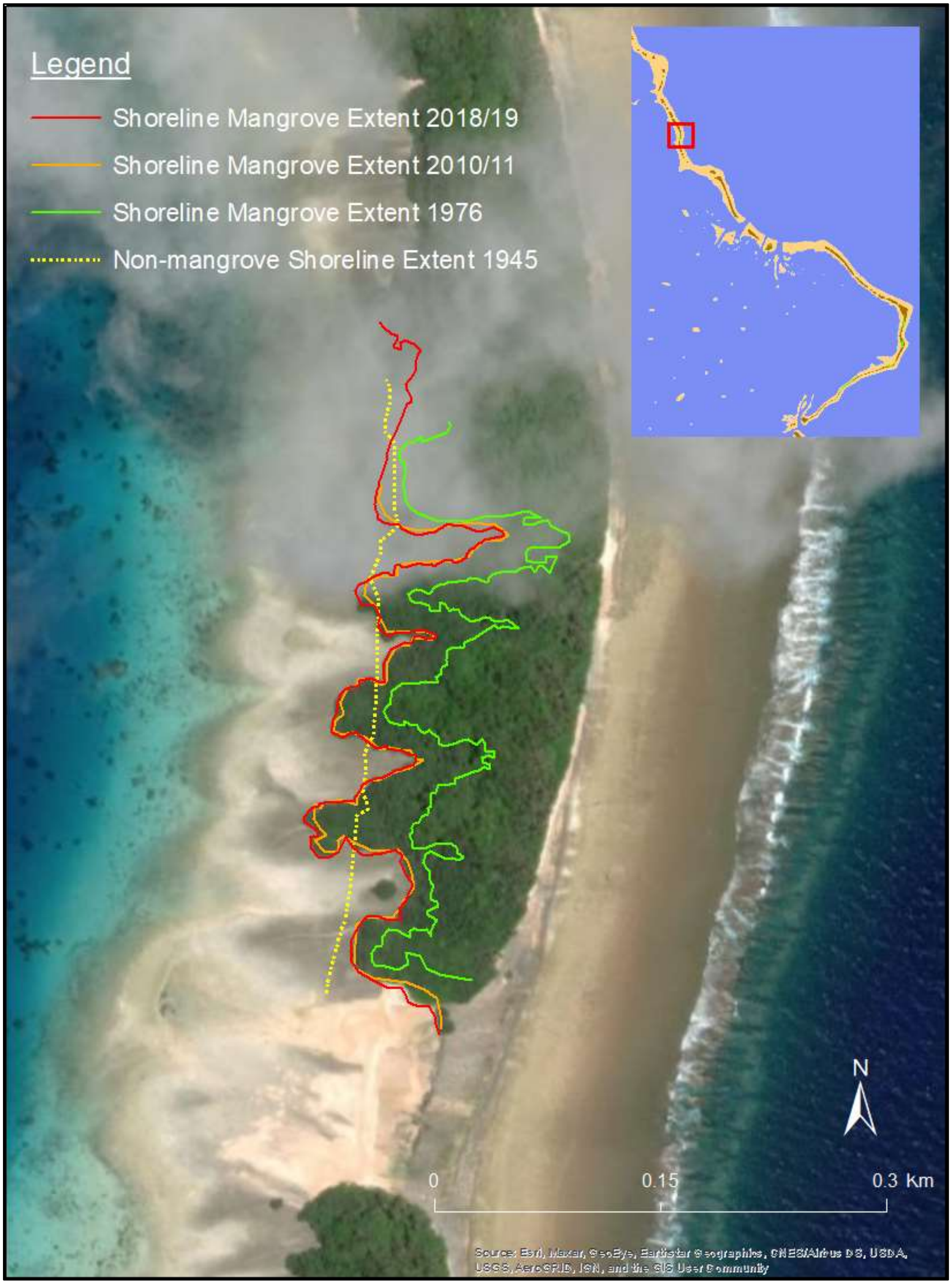

Fig S10 Mangrove shoreline change, Medyai

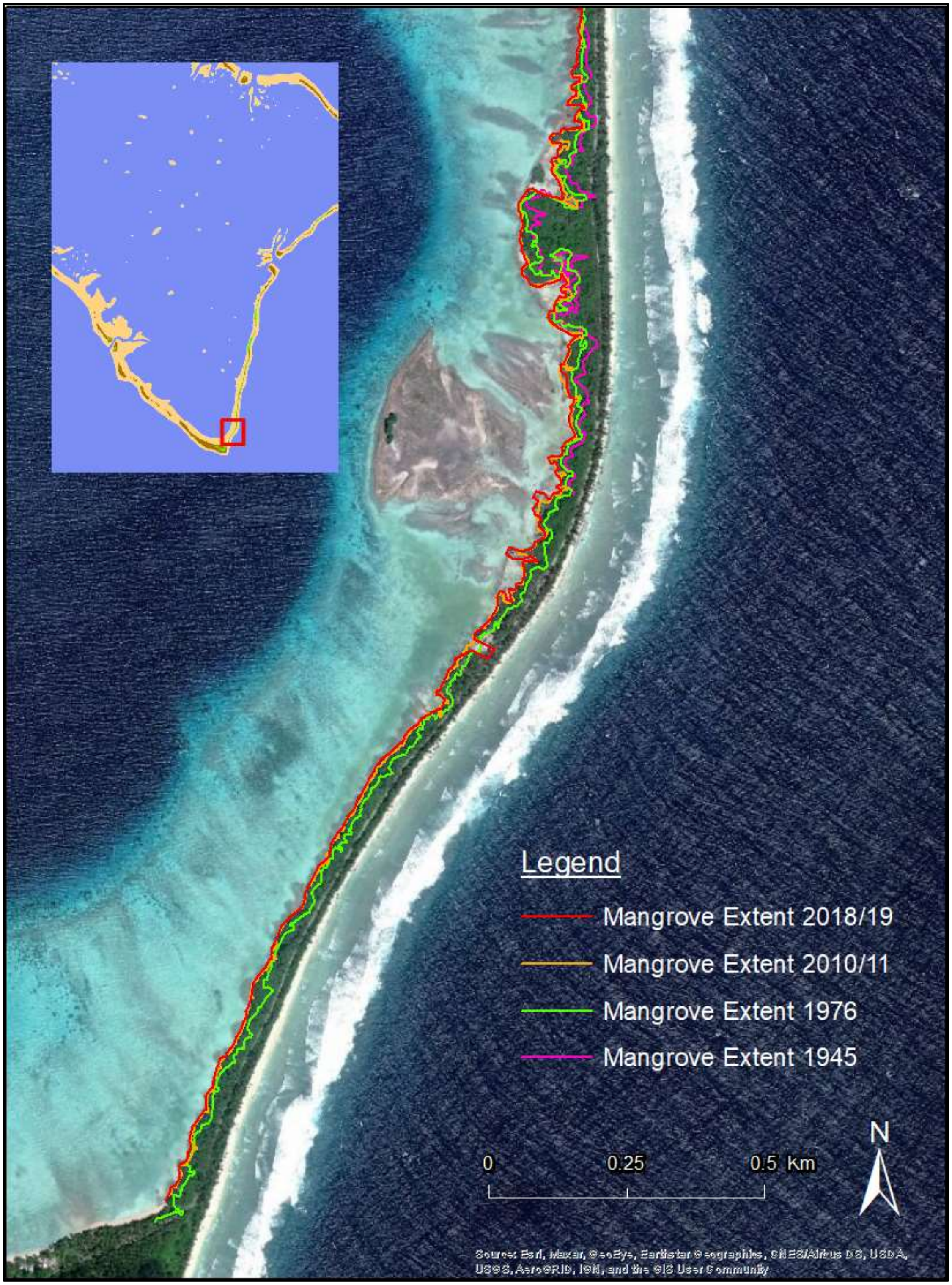

Fig S11 Mangrove shoreline change, Jaluit Jaluit 1

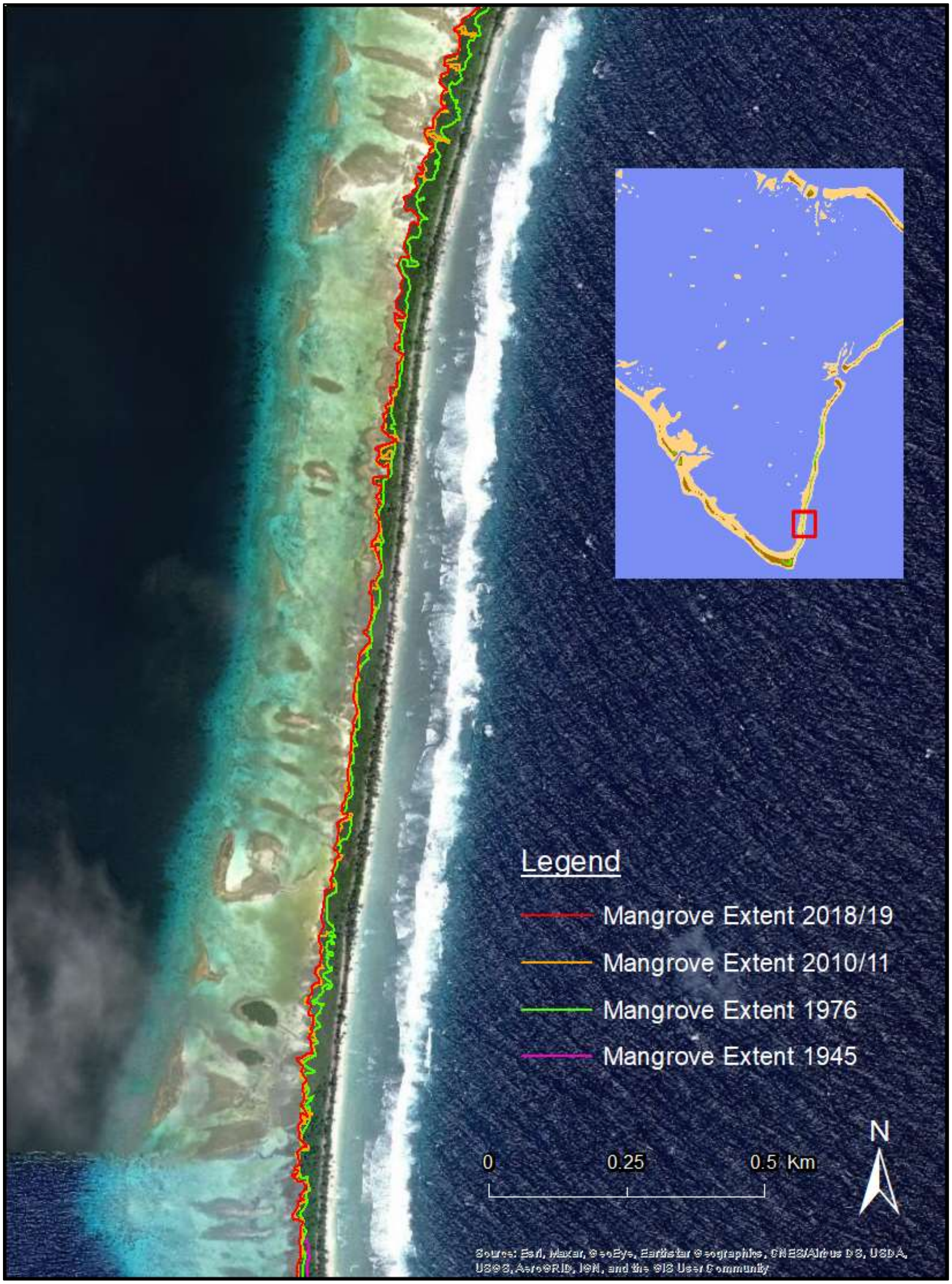

Fig S12 Mangrove shoreline change, Jaluit Jaluit 2

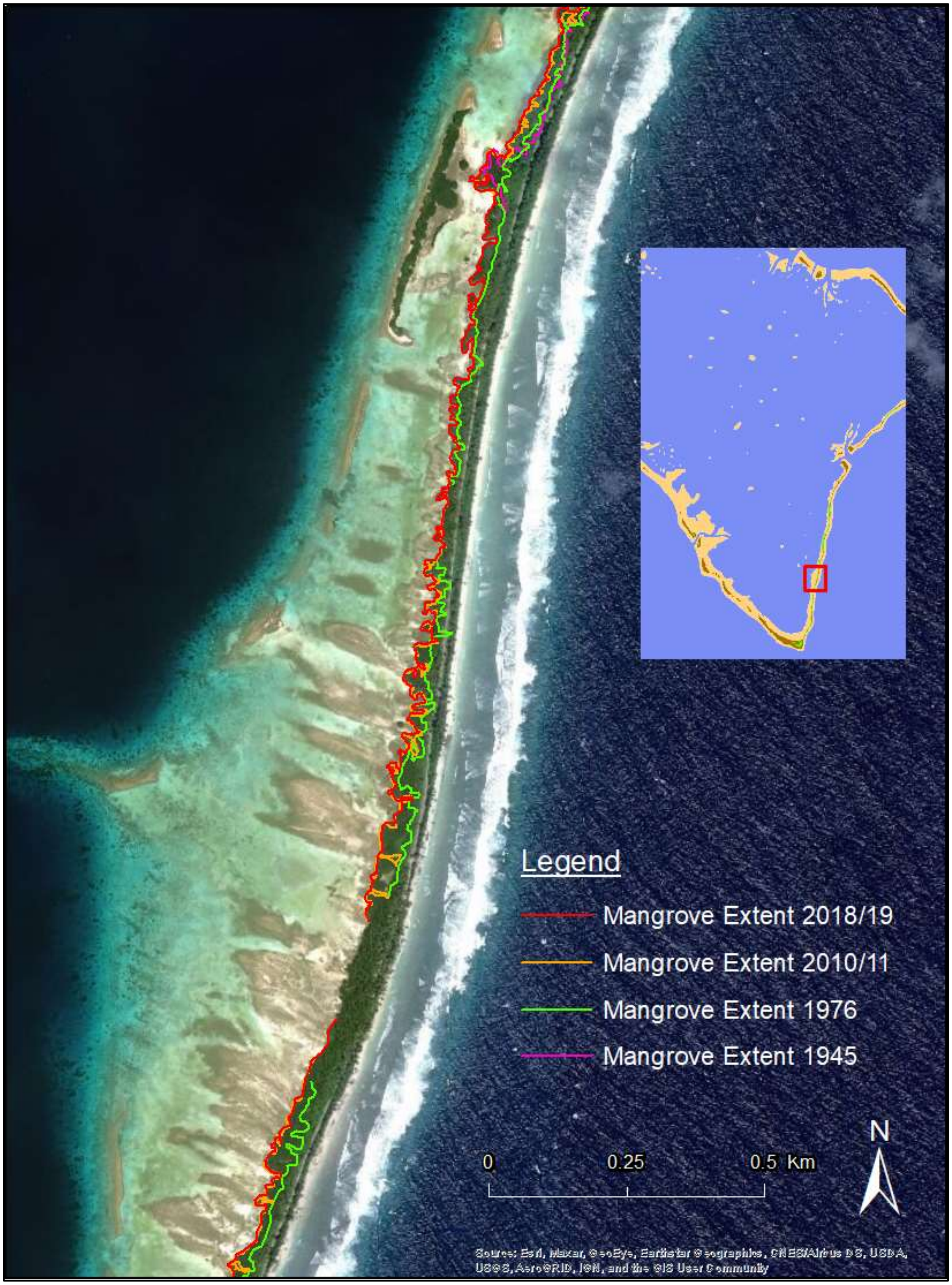

Fig S13 Mangrove shoreline change, Jaluit Jaluit 3

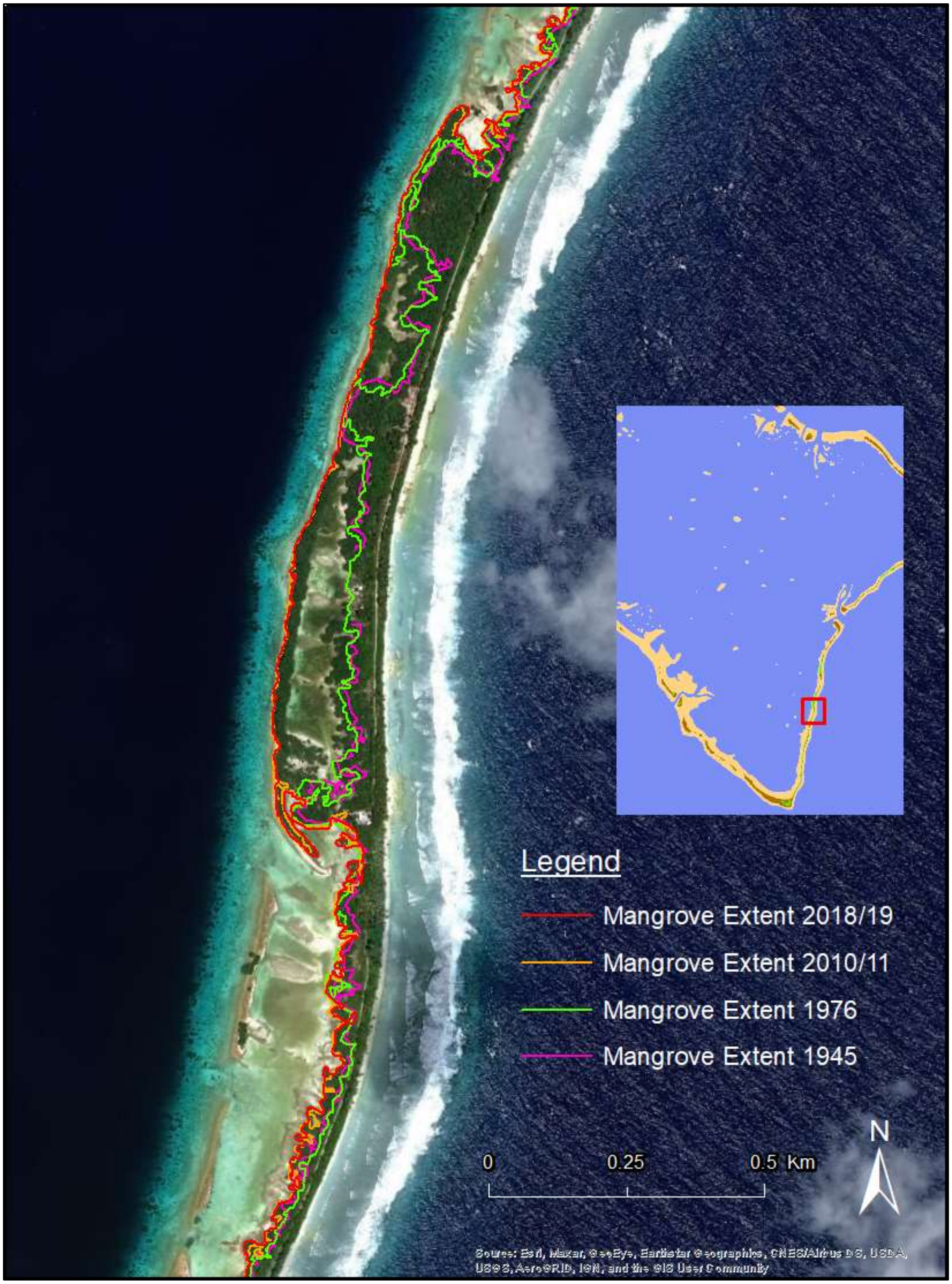

Fig S14 Mangrove shoreline change, Jaluit Jaluit 4

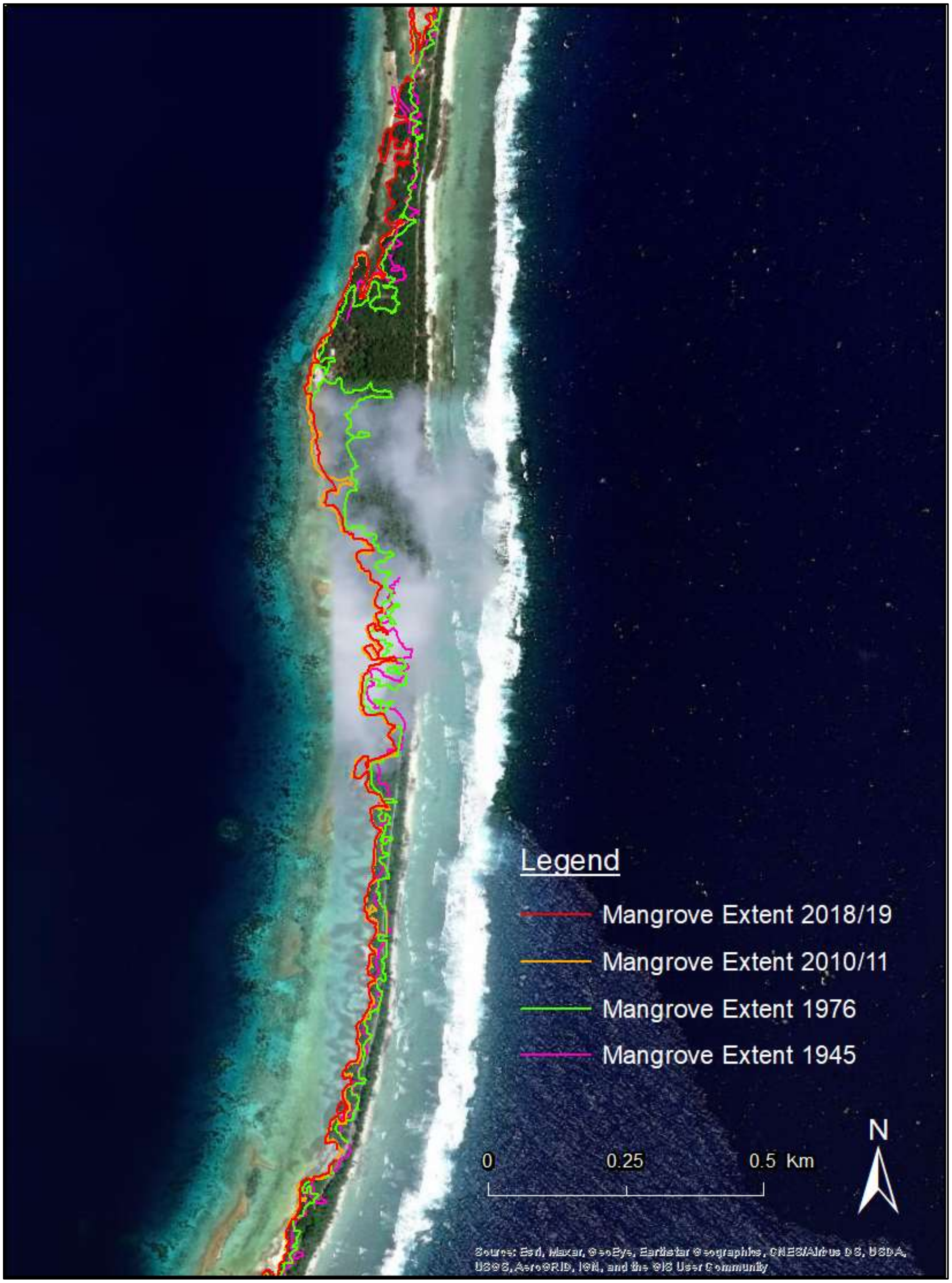

Fig S15 Mangrove shoreline change, Jaluit Jaluit 5

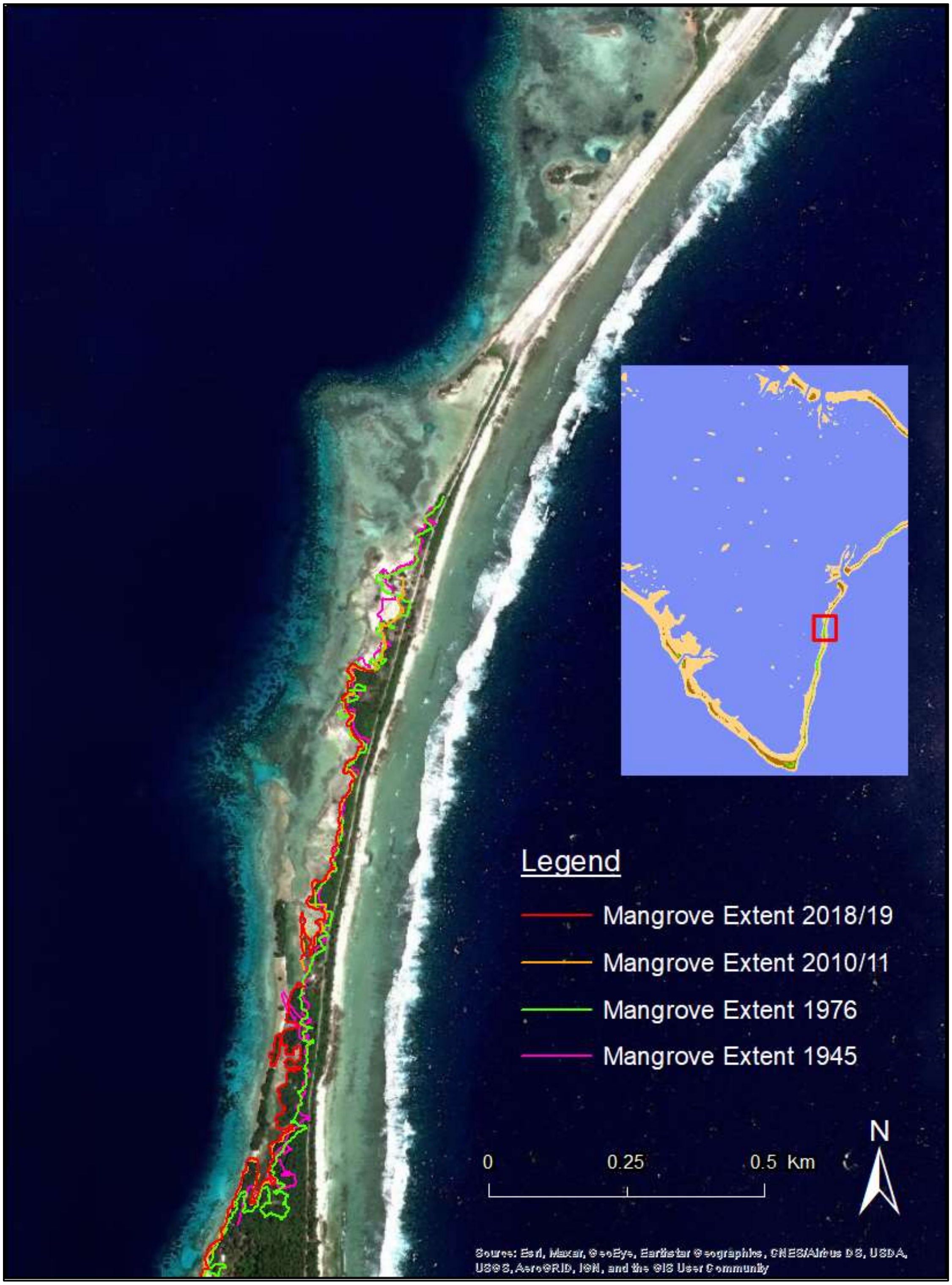

Fig S16 Mangrove shoreline change, Jaluit Jaluit 6

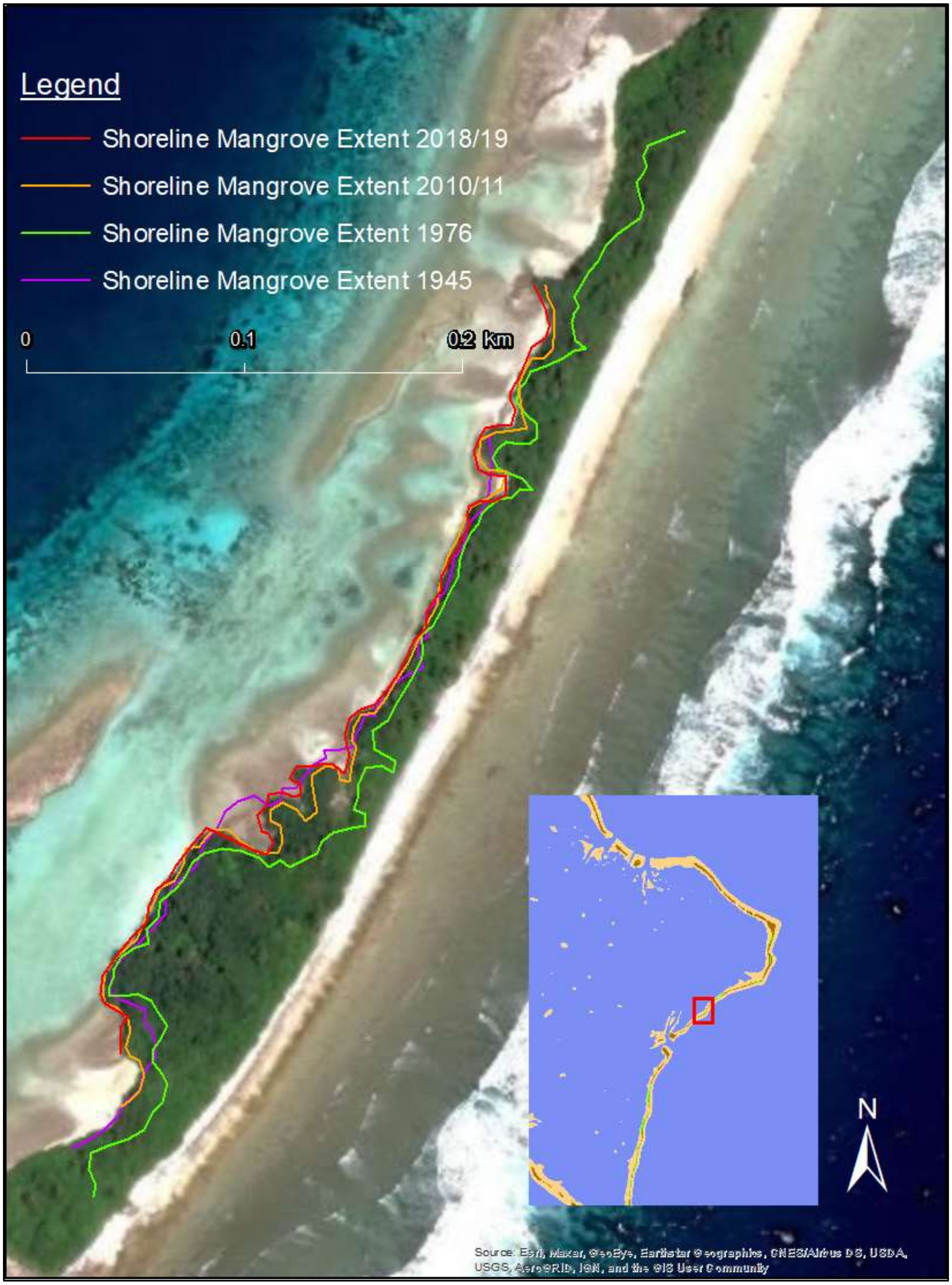

Fig S17 Mangrove shoreline change, Aineman 1

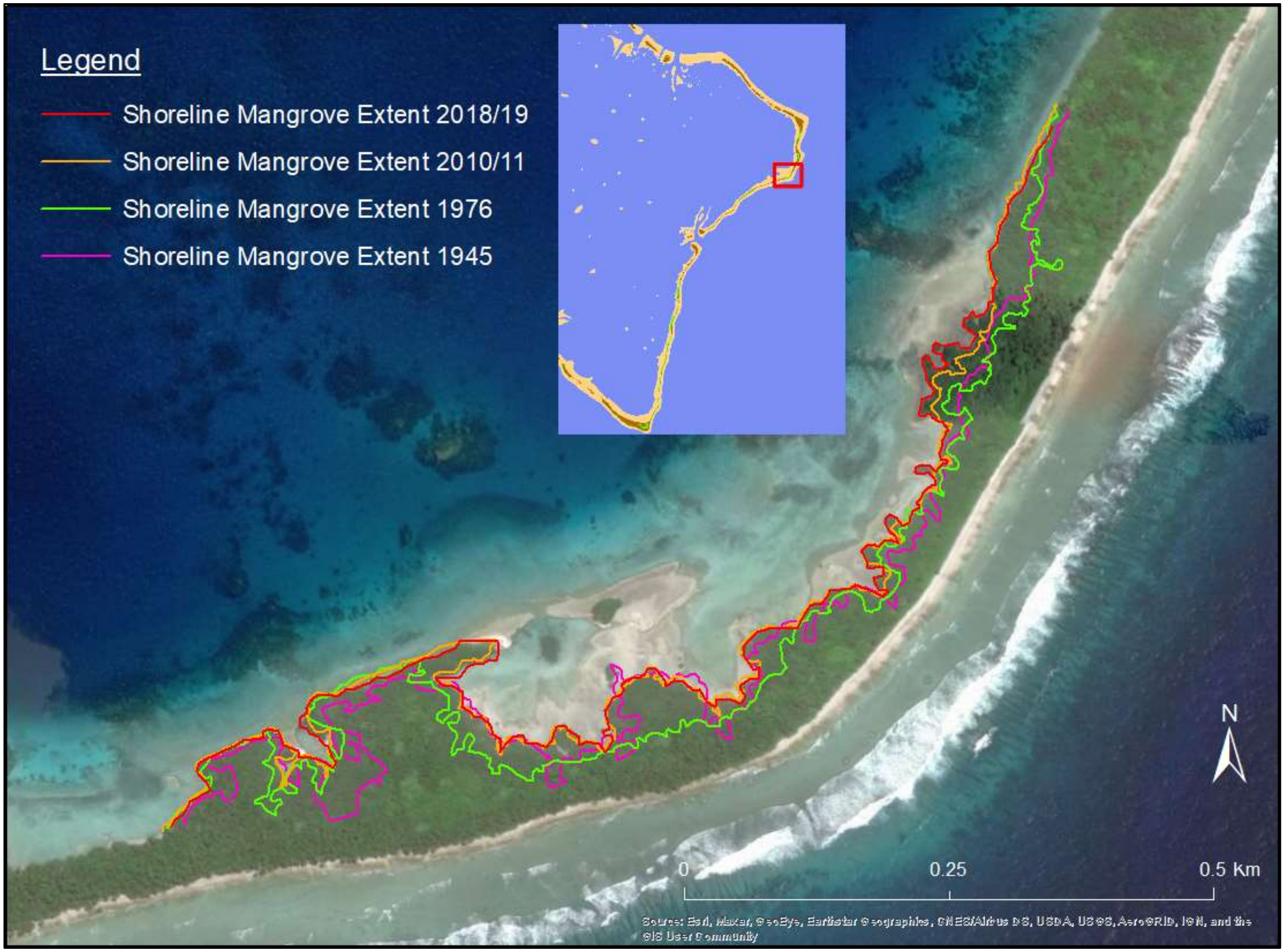

Fig S18 Mangrove shoreline change, Aineman 2

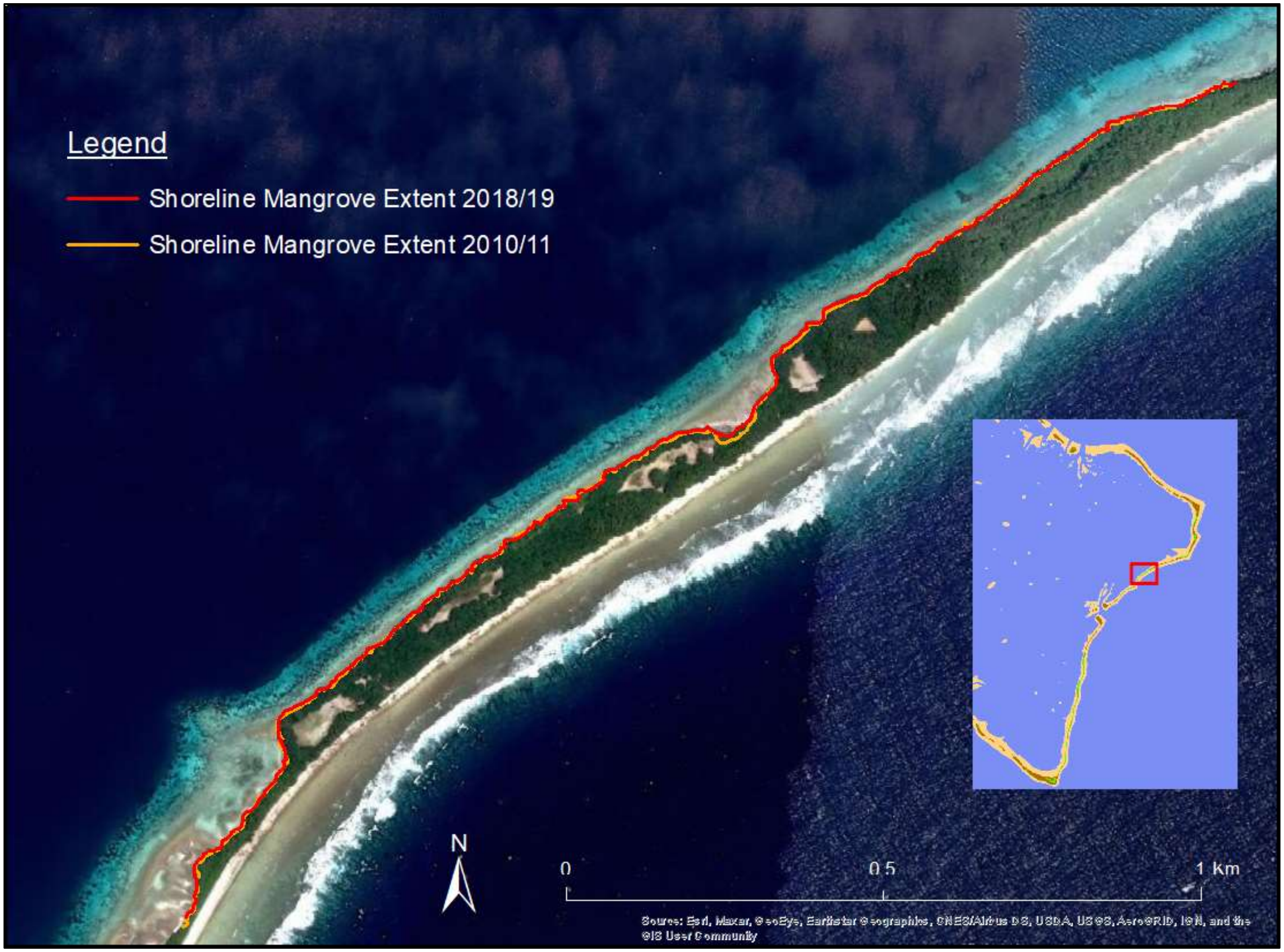

Fig S19 Mangrove shoreline change, Aineman 3

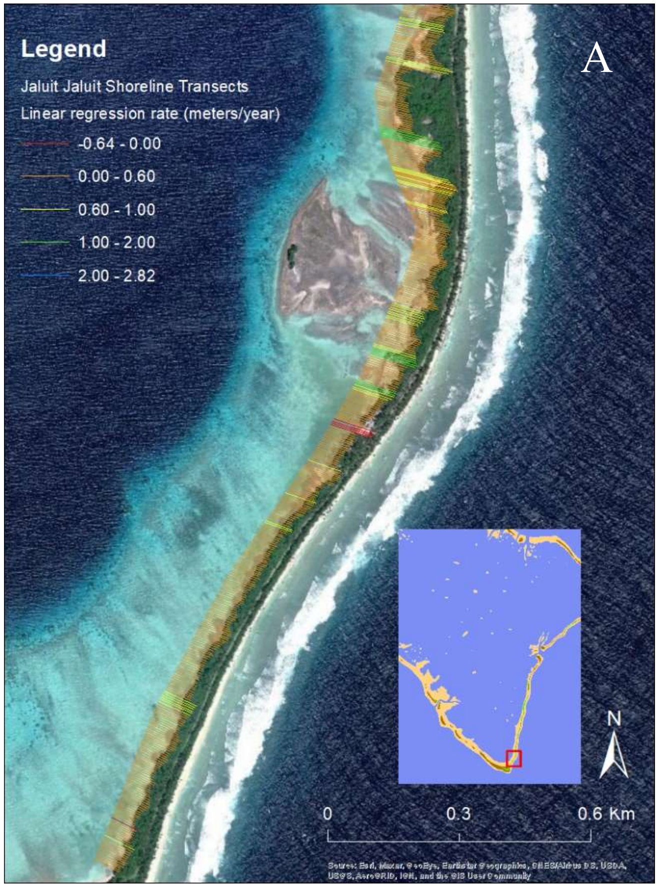
Legend
Jaluit Jaluit Shoreline Transects
Linear regression rate (meters/year)
-0.64 - 0.00
0.00 - 0.60
0.60 - 1.00
1.00 - 2.00
2.00 - 2.82
A
N
0
0.3
0.6 Km

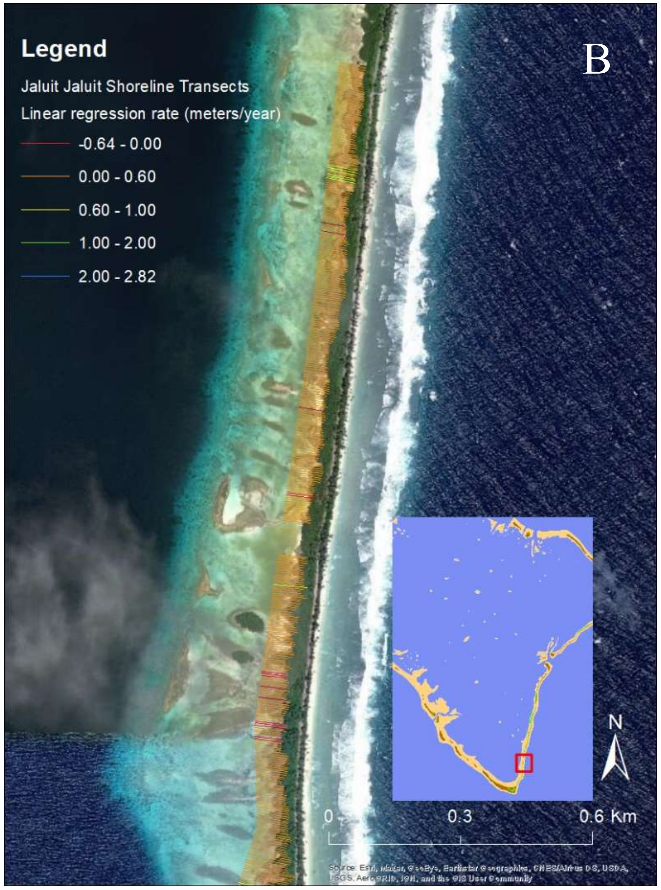
Legend
Jaluit Jaluit Shoreline Transects
Linear regression rate (meters/year)
-0.64 - 0.00
0.00 - 0.60
0.60 - 1.00
1.00 - 2.00
2.00 - 2.82
B
N
0
0.3
0.6 Km

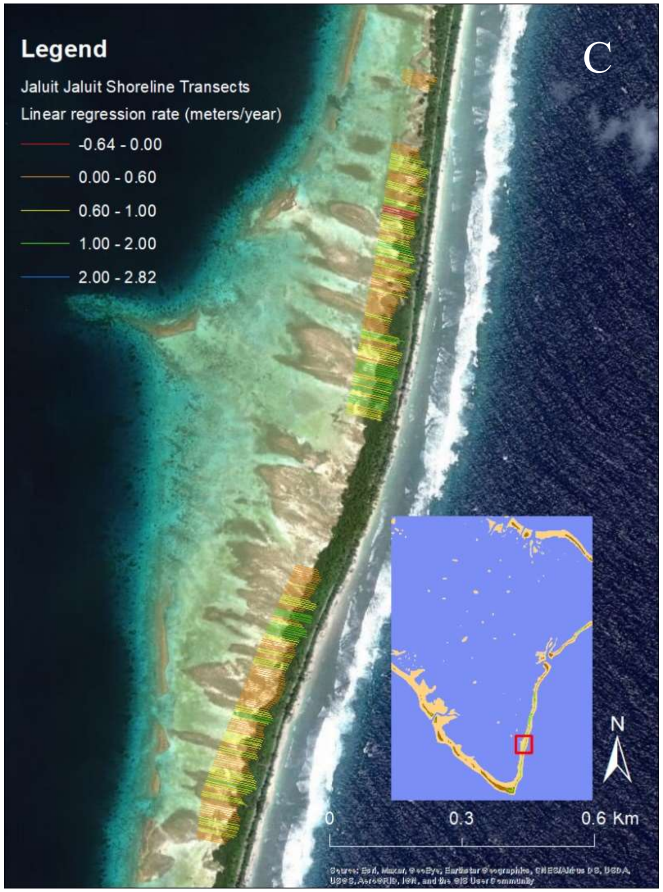
Legend
Jaluit Jaluit Shoreline Transects
Linear regression rate (meters/year)
-0.64 - 0.00
0.00 - 0.60
0.60 - 1.00
1.00 - 2.00
2.00 - 2.82
C
N
0
0.3
0.6 Km

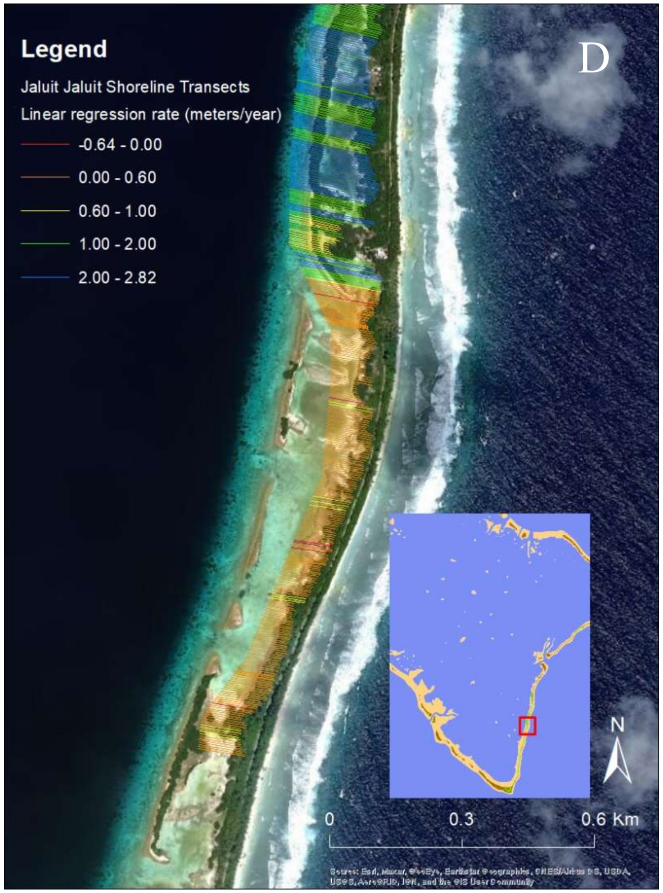
Legend
Jaluit Jaluit Shoreline Transects
Linear regression rate (meters/year)
-0.64 - 0.00
0.00 - 0.60
0.60 - 1.00
1.00 - 2.00
2.00 - 2.82
D
N
0
0.3
0.6 Km

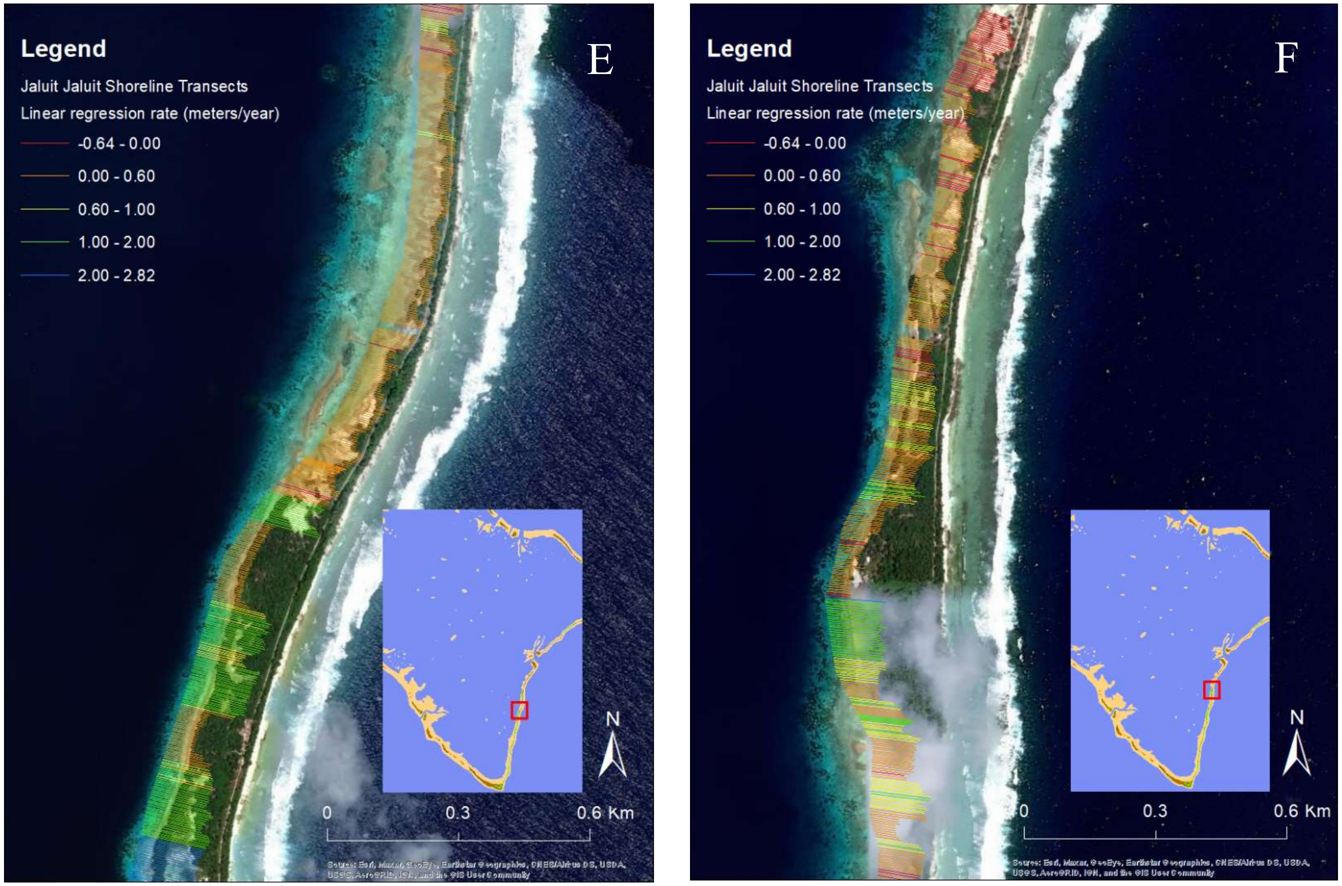

Fig. S20 Lagoon mangrove DSAS results from Jaluit Jaluit A. most southerly to F. most northerly

Table S1 Inland mangrove areas (m^2) spatial change over time.

Inland mangrove wetlands	Area 2018/19 (m^2)	Area 2010-11 (m^2)	Area 1976 (m^2)	Area 1945 (m^2)
Ae	49,080	48,430	41,715	37,749
Emidj_1	11,970	19,636	26,476	-
Emidj_2	69,878	73,127	26,724	17,129
Emidj_3	24,000	24,239	78,122	-
Ertok (Classified as coastal mangroves)	14,816	13,658	11,179	15,585
Ewo	9,497	10,059	25,926	29,638
Imiej_1	74,059	6,310	84,868	48,196
Imiej_2	7,307	65,634	-	-
Jaluit_1	188,702	161,797	195,295	237,689
Jaluit_2	5,453	4,781	3,771	87,281
Jaluit_3	10,579	10,407	9,498	29,869
Kinajon_1	9,963	9,922	8,945	7,997
Kinajon_2	9,688	9,320	8,570	3,729
Kinajon_3	-	-	-	2,912
Mejrirok	33,835	30,249	23,051	24,272
Pinglep_1	4,029	3,162	3,127	4,426
Pinglep_2	10,382	10,249	11,537	7,776
**Total area m^2**	533,238	500,980	558,804	554,248
**Total area of lakes and non-mangrove areas**	20,747	59,073	146,718	109,771
**Total area m^2 without lakes and non-mangrove areas (including Ertok)**	497,675 (512,491)	428,249 (441,907)	400,907 (412,086)	428,892 (444,477)
Note: - indicates this area of mangroves either was not present or was part of another mangroves area. Ertok area is removed from Table 4 calculations.				

Datasets generated during the DSAS

File name: DSAS_Summary_Transect_Jaluit_20201004_144802.txt

Timestamp of rate calculation: 10/04/2020 14:52:43

DSAS version: 5.0.20200527.0200

ArcGIS version: 10.8

Rate types run: SCE, NSM, EPR, LRR, WLR

Baseline layer: Baseline_Jaluit

Shoreline layer: Shoreline_Jaluit

Shoreline dates used: 3/18/1945, 1/8/1976, 4/3/2011, 12/12/2018

Shoreline threshold: 3

Confidence Interval (CI) selected: 90

Default Uncertainty: 10

Transect spacing length: 5

Smoothing distance: 2500

Coordinate system: WGS_1984_UTM_Zone_59N

Is bias applied: NO

All rates reported are in meters/year, distance values are in meters.

DISTANCE: SCE (Shoreline Change Envelope, m)

SCE OVERALL AVERAGES:

total number of transects: 2519

average distance: 28.85

maximum distance: 169.66

maximum distance transect ID: 1603

minimum distance: 0.16

minimum distance transect ID: 1024

DISTANCE: NSM (Net Shoreline Movement, m)

NSM OVERALL AVERAGES:

total number of transects: 2519

average distance: 25.28

number of transects with negative distance: 138

percent of all transects that have a negative distance: 5.48%

maximum negative distance: -42.34

maximum negative distance transect ID: 2518

average of all negative distances: -8.77

number of transects with positive distance: 2381

percent of all transects that have a positive distance: 94.52%

maximum positive distance: 167.62

maximum positive distance transect ID: 1603

average of all positive distances: 27.26

RATE: EPR (End Point Rate, m/yr)

EPR OVERALL AVERAGES:

total number of transects: 2519

average rate: 0.44

average of the confidence intervals associated with rates: 0.29

reduced n (number of independent transects): 31

uncertainty of the average rate using reduced n: 0.05

average rate with reduced n uncertainty: 0.44 +/- 0.05

number of erosional transects: 138

percent of all transects that are erosional: 5.48%

percent of all transects that have statistically significant erosion: 1.07%

maximum value erosion: -0.64

maximum value erosion transect ID: 2518

average of all erosional rates: -0.18

number of accretional transects: 2381

percent of all transects that are accretional: 94.52%

percent of all transects that have statistically significant accretion: 59.35%

maximum value accretion: 2.69

maximum value accretion transect ID: 1203

average of all accretional rates: 0.47

RATE: LRR (Linear Regression Rate, m/yr)

LRR OVERALL AVERAGES:

total number of transects: 2272

average rate: 0.49

average of the confidence intervals associated with rates: 0.61

reduced n (number of independent transects): 61

uncertainty of the average rate using reduced n: 0.08

average rate with reduced n uncertainty: 0.49 +/- 0.08

number of erosional transects: 98

percent of all transects that are erosional: 4.31%

percent of all transects that have statistically significant erosion: 0.35%

maximum value erosion: -0.64

maximum value erosion transect ID: 2518

average of all erosional rates: -0.16

number of accretional transects: 2174

percent of all transects that are accretional: 95.69%

percent of all transects that have statistically significant accretion: 50.04%

maximum value accretion: 2.81

maximum value accretion transect ID: 1670

average of all accretional rates: 0.51

RATE: WLR (Weighted Linear Regression, m/yr)

WLR OVERALL AVERAGES:

total number of transects: 2272

average rate: 0.49

average of the confidence intervals associated with rates: 0.61

reduced n (number of independent transects): 61

uncertainty of the average rate using reduced n: 0.08

average rate with reduced n uncertainty: 0.49 +/- 0.08

number of erosional transects: 98

percent of all transects that are erosional: 4.31%

percent of all transects that have statistically significant erosion: 0.35%

maximum value erosion: -0.64

maximum value erosion transect ID: 2518

average of all erosional rates: -0.16

number of accretional transects: 2174

percent of all transects that are accretional: 95.69%

percent of all transects that have statistically significant accretion: 50.04%

maximum value accretion: 2.81

maximum value accretion transect ID: 1670

average of all accretional rates: 0.51

File name: DSAS_Summary_Transect_Medyai2_20200921_234413.txt

Timestamp of rate calculation: 09/21/2020 23:44:45

DSAS version: 5.0.20200527.0200

ArcGIS version: 10.8

Rate types run: SCE, NSM, EPR, LRR, WLR

Baseline layer: Baseline_Medyai

Shoreline layer: Shorelines_Medyai

Shoreline dates used: 1/8/1976, 12/17/2010, 7/1/2019

Shoreline threshold: 3

Confidence Interval (CI) selected: 90

Default Uncertainty: 10

Transect spacing length: 5

Smoothing distance: 2500

Coordinate system: WGS_1984_UTM_Zone_59N

Is bias applied: NO

All rates reported are in meters/year, distance values are in meters.

DISTANCE: SCE (Shoreline Change Envelope, m)

SCE OVERALL AVERAGES:

total number of transects: 65

average distance: 44.72

maximum distance: 122.41

maximum distance transect ID: 28

minimum distance: 13.62

minimum distance transect ID: 50

DISTANCE: NSM (Net Shoreline Movement, m)

NSM OVERALL AVERAGES:

total number of transects: 65

average distance: 44.35

number of transects with negative distance: 0

percent of all transects that have a negative distance: 0%

maximum negative distance:

maximum negative distance transect ID:

average of all negative distances:

number of transects with positive distance: 65

percent of all transects that have a positive distance: 100%

maximum positive distance: 122.41

maximum positive distance transect ID: 28

average of all positive distances: 44.35

RATE: EPR (End Point Rate, m/yr)

EPR OVERALL AVERAGES:

total number of transects: 65

average rate: 1.02

average of the confidence intervals associated with rates: 0.33

reduced n (number of independent transects): 1

uncertainty of the average rate using reduced n: 0.33

average rate with reduced n uncertainty: 1.02 +/- 0.33

number of erosional transects: 0

percent of all transects that are erosional: 0%

percent of all transects that have statistically significant erosion: 0%

maximum value erosion:

maximum value erosion transect ID:

average of all erosional rates:

number of accretional transects: 65

percent of all transects that are accretional: 100%

percent of all transects that have statistically significant accretion: 93.85%

maximum value accretion: 2.82

maximum value accretion transect ID: 28

average of all accretional rates: 1.02

RATE: LRR (Linear Regression Rate, m/yr)

LRR OVERALL AVERAGES:

total number of transects: 65

average rate: 1.07

average of the confidence intervals associated with rates: 1.07

reduced n (number of independent transects): 20

uncertainty of the average rate using reduced n: 0.24

average rate with reduced n uncertainty: 1.07 +/- 0.24

number of erosional transects: 0

percent of all transects that are erosional: 0%

percent of all transects that have statistically significant erosion: 0%

maximum value erosion:

maximum value erosion transect ID:

average of all erosional rates:

number of accretional transects: 65

percent of all transects that are accretional: 100%

percent of all transects that have statistically significant accretion: 61.54%

maximum value accretion: 2.96

maximum value accretion transect ID: 28

average of all accretional rates: 1.07

RATE: WLR (Weighted Linear Regression, m/yr)

WLR OVERALL AVERAGES:

total number of transects: 65

average rate: 1.07

average of the confidence intervals associated with rates: 1.07

reduced n (number of independent transects): 20

uncertainty of the average rate using reduced n: 0.24

average rate with reduced n uncertainty: 1.07 +/- 0.24

number of erosional transects: 0

percent of all transects that are erosional: 0%

percent of all transects that have statistically significant erosion: 0%

maximum value erosion:

maximum value erosion transect ID:

average of all erosional rates:

number of accretional transects: 65

percent of all transects that are accretional: 100%

percent of all transects that have statistically significant accretion: 61.54%

maximum value accretion: 2.96

maximum value accretion transect ID: 28

average of all accretional rates: 1.07

File name: DSAS_Summary_Transect_Aineman1_20201004_125935.txt

Timestamp of rate calculation: 10/04/2020 12:59:42

DSAS version: 5.0.20200527.0200

ArcGIS version: 10.8

Rate types run: SCE, NSM, EPR, LRR, WLR

Baseline layer: Baseline_Aineman_1

Shoreline layer: Shoreline_Aineman_1

Shoreline dates used: 3/18/1946, 1/8/1976, 4/3/2011, 12/12/2018

Shoreline threshold: 3

Confidence Interval (CI) selected: 90

Default Uncertainty: 10

Transect spacing length: 5

Smoothing distance: 2500

Coordinate system: WGS_1984_UTM_Zone_59N

Is bias applied: NO

All rates reported are in meters/year, distance values are in meters.

DISTANCE: SCE (Shoreline Change Envelope, m)

SCE OVERALL AVERAGES:

total number of transects: 95

average distance: 12.9

maximum distance: 44.42

maximum distance transect ID: 40

minimum distance: 2.39

minimum distance transect ID: 22

DISTANCE: NSM (Net Shoreline Movement, m)

NSM OVERALL AVERAGES:

total number of transects: 95

average distance: 2.69

number of transects with negative distance: 22

percent of all transects that have a negative distance: 23.16%

maximum negative distance: -25.98

maximum negative distance transect ID: 38

average of all negative distances: -8.57

number of transects with positive distance: 73

percent of all transects that have a positive distance: 76.84%

maximum positive distance: 23.18

maximum positive distance transect ID: 19

average of all positive distances: 6.09

RATE: EPR (End Point Rate, m/yr)

EPR OVERALL AVERAGES:

total number of transects: 95

average rate: 0.04

average of the confidence intervals associated with rates: 0.24

reduced n (number of independent transects): 8

uncertainty of the average rate using reduced n: 0.08

average rate with reduced n uncertainty: 0.04 +/- 0.08

number of erosional transects: 22

percent of all transects that are erosional: 23.16%

percent of all transects that have statistically significant erosion: 2.11%

maximum value erosion: -0.45

maximum value erosion transect ID: 10

average of all erosional rates: -0.18

number of accretional transects: 73

percent of all transects that are accretional: 76.84%

percent of all transects that have statistically significant accretion: 7.37%

maximum value accretion: 0.46

maximum value accretion transect ID: 98

average of all accretional rates: 0.11

RATE: LRR (Linear Regression Rate, m/yr)

LRR OVERALL AVERAGES:

total number of transects: 87

average rate: 0.08

average of the confidence intervals associated with rates: 0.36

reduced n (number of independent transects): 9

uncertainty of the average rate using reduced n: 0.12

average rate with reduced n uncertainty: 0.08 +/- 0.12

number of erosional transects: 10

percent of all transects that are erosional: 11.49%

percent of all transects that have statistically significant erosion: 0%

maximum value erosion: -0.3

maximum value erosion transect ID: 38

average of all erosional rates: -0.1

number of accretional transects: 77

percent of all transects that are accretional: 88.51%

percent of all transects that have statistically significant accretion: 25.29%

maximum value accretion: 0.4

maximum value accretion transect ID: 19

average of all accretional rates: 0.1

RATE: WLR (Weighted Linear Regression, m/yr)

WLR OVERALL AVERAGES:

total number of transects: 87

average rate: 0.08

average of the confidence intervals associated with rates: 0.36

reduced n (number of independent transects): 9

uncertainty of the average rate using reduced n: 0.12

average rate with reduced n uncertainty: 0.08 +/- 0.12

number of erosional transects: 10

percent of all transects that are erosional: 11.49%

percent of all transects that have statistically significant erosion: 0%

maximum value erosion: -0.3

maximum value erosion transect ID: 38

average of all erosional rates: -0.1

number of accretional transects: 77

percent of all transects that are accretional: 88.51%

percent of all transects that have statistically significant accretion: 25.29%

maximum value accretion: 0.4

maximum value accretion transect ID: 19

average of all accretional rates: 0.1

File name: DSAS_Summary_Transect_Aineman3_20201004_110812.txt

Timestamp of rate calculation: 10/04/2020 11:08:53

DSAS version: 5.0.20200527.0200

ArcGIS version: 10.8

Rate types run: SCE, NSM, EPR, LRR, WLR

Baseline layer: baseline_Aineman3

Shoreline layer: Aineman_3_Shoreline

Shoreline dates used: 3/18/1945, 1/8/1976, 10/3/2010, 5/27/2018

Shoreline threshold: 4

Confidence Interval (CI) selected: 90

Default Uncertainty: 10

Transect spacing length: 5

Smoothing distance: 2500

Coordinate system: WGS_1984_UTM_Zone_59N

Is bias applied: NO

All rates reported are in meters/year, distance values are in meters.

DISTANCE: SCE (Shoreline Change Envelope, m)

SCE OVERALL AVERAGES:

total number of transects: 243

average distance: 36.8

maximum distance: 154.91

maximum distance transect ID: 63

minimum distance: 2.95

minimum distance transect ID: 2

DISTANCE: NSM (Net Shoreline Movement, m)

NSM OVERALL AVERAGES:

total number of transects: 243

average distance: 21.77

number of transects with negative distance: 29

percent of all transects that have a negative distance: 11.93%

maximum negative distance: -37.06

maximum negative distance transect ID: 112

average of all negative distances: -7.65

number of transects with positive distance: 214

percent of all transects that have a positive distance: 88.07%

maximum positive distance: 150.7

maximum positive distance transect ID: 70

average of all positive distances: 25.76

RATE: EPR (End Point Rate, m/yr)

EPR OVERALL AVERAGES:

total number of transects: 243

average rate: 0.3

average of the confidence intervals associated with rates: 0.2

reduced n (number of independent transects): 1

uncertainty of the average rate using reduced n: 0.2

average rate with reduced n uncertainty: 0.3 +/- 0.2

number of erosional transects: 29

percent of all transects that are erosional: 11.93%

percent of all transects that have statistically significant erosion: 2.47%

maximum value erosion: -0.51

maximum value erosion transect ID: 112

average of all erosional rates: -0.11

number of accretional transects: 214

percent of all transects that are accretional: 88.07%

percent of all transects that have statistically significant accretion: 46.5%

maximum value accretion: 2.06

maximum value accretion transect ID: 70

average of all accretional rates: 0.35

RATE: LRR (Linear Regression Rate, m/yr)

LRR OVERALL AVERAGES:

total number of transects: 242

average rate: 0.36

average of the confidence intervals associated with rates: 0.68

reduced n (number of independent transects): 10

uncertainty of the average rate using reduced n: 0.21

average rate with reduced n uncertainty: 0.36 +/- 0.21

number of erosional transects: 13

percent of all transects that are erosional: 5.37%

percent of all transects that have statistically significant erosion: 0%

maximum value erosion: -0.4

maximum value erosion transect ID: 112

average of all erosional rates: -0.08

number of accretional transects: 229

percent of all transects that are accretional: 94.63%

percent of all transects that have statistically significant accretion: 29.75%

maximum value accretion: 2.37

maximum value accretion transect ID: 73

average of all accretional rates: 0.38

RATE: WLR (Weighted Linear Regression, m/yr)

WLR OVERALL AVERAGES:

total number of transects: 242

average rate: 0.36

average of the confidence intervals associated with rates: 0.68

reduced n (number of independent transects): 10

uncertainty of the average rate using reduced n: 0.21

average rate with reduced n uncertainty: 0.36 +/- 0.21

number of erosional transects: 13

percent of all transects that are erosional: 5.37%

percent of all transects that have statistically significant erosion: 0%

maximum value erosion: -0.4

maximum value erosion transect ID: 112

average of all erosional rates: -0.08

number of accretional transects: 229

percent of all transects that are accretional: 94.63%

percent of all transects that have statistically significant accretion: 29.75%

maximum value accretion: 2.37

maximum value accretion transect ID: 73

average of all accretional rates: 0.38
